# Genome-Wide Identification of Sultr Genes in *Malus domestica* and Low Sulfur-Induced *MhSultr3;1a* to Increase Cysteine-Improving Growth

**DOI:** 10.3389/fpls.2021.748242

**Published:** 2021-10-11

**Authors:** Mi Xun, Jianfei Song, Junyuan Shi, Jiaqi Li, Yujia Shi, Junhong Yan, Weiwei Zhang, Hongqiang Yang

**Affiliations:** State Key Laboratory of Crop Biology, College of Horticulture Science and Engineering, Shandong Agricultural University, Tai'an, China

**Keywords:** genome-wide identification, *Malus*, Sultr gene family, low sulfur, sulfate, cysteine

## Abstract

Sulfur is an essential nutrient for plant growth and development. Sulfate transporters (Sultrs) are critical for sulfate (${\text{SO}}_{4}^{2-}$) uptake from the soil by the roots in higher plants. However, knowledge about Sultrs in apples (*Malus domestica*) is scarce. Here, nine putative *MdSultrs* were identified and classified into two groups according to the their phylogenetic relationships, gene structures, and conserved motifs. Various cis-regulatory elements related to abiotic stress and plant hormone responsiveness were found in the promoter regions of *MdSultrs*. These *MdSultrs* exhibited tissue-specific expression patterns and responded to low sulfur (S), abscisic acid (ABA), indole-3-acetic acid (IAA), and methyl jasmonate (MeJA), wherein *MdSultr3;1a* was especially expressed in the roots and induced by low S. The uptake of ${\text{SO}}_{4}^{2-}$ in cultivated apples depends on the roots of its rootstock, and *MhSultr3;1a* was isolated from *Malus hupehensis* roots used as a rootstock. *MhSultr3*;1a shared 99.85% homology with *MdSultr3;1a* and localized on the plasma membrane and nucleus membrane. Further function characterization revealed that *MhSultr3;1a* complemented an ${\text{SO}}_{4}^{2-}$ transport-deficient yeast mutant and improved the growth of yeast and apple calli under low S conditions. The *MhSultr3;1a*-overexpressing apple calli had a higher fresh weight compared with the wild type (WT) under a low-S treatment because of the increased ${\text{SO}}_{4}^{2-}$ and cysteine (Cys) content. These results demonstrate that *MhSultr3;1a* may increase the content of ${\text{SO}}_{4}^{2-}$ and Cys to meet the demands of S-containing compounds and improve their growth under S-limiting conditions.

## Introduction

Sulfur is an essential macronutrient for plant growth and development. It is required for the biosynthesis of sulfur (S)-containing amino acids, namely, cysteine (Cys) and methionine (Met) (Aarabi et al., 2020). Both Cys and Met serve as building blocks for proteins and are precursors for the synthesis of hydrogen sulfide (H_2_S), glutathione (GSH), phytochelatins (PCs), and secondary metabolites (Romero et al., 2014). These compounds are involved in various biological processes, such as protein synthesis and metabolism, photosynthesis, and heavy-metal detoxification (Kopriva et al., 2019; Chen et al., 2020c; Shi et al., 2020). An S deficiency decreases the proportion of S-containing amino acids in plants, limits protein synthesis, and reduces photosynthetic activity, thereby resulting in the loss of crop yield and quality (Aarabi et al., 2020). In higher plants, sulfate (${\text{SO}}_{4}^{2-}$), the main form of inorganic S utilized by plants, is obtained from the soil through a hydron (H^+^)-dependent cotransport process that relies on sulfate transporters (Sultrs), followed by activation and reduction reactions to form Cys for further metabolic processes (Takahashi, 2019).

Sulfate transporters, encoded by multigene families, are critical for the uptake and intracellular, cell-to-cell, and long-distance transport of ${\text{SO}}_{4}^{2-}$ in plants (Buchner et al., 2004b; Takahashi, 2019). To date, Sultrs have been identified in several plant species, such as Arabidopsis (*Arabidopsis thaliana*) (Takahashi, 2010), wheat (Buchner et al., 2010), rice (Kumar et al., 2011), soybeans (Ding et al., 2016), potatoes (Vatansever et al., 2016), maize (Huang et al., 2018), and sorghum (Akbudak et al., 2018). All Sultrs contain 12 transmembrane domains (TMDs) and 1 Sultr anti-sigma (STAS) domain in the C-terminal region (Takahashi, 2019). The STAS domain plays an important role in the proper localization, biosynthesis/stability, and catalytic characteristics of Sultrs, and participates in regulating the activity of O-acetyl-serine (thiol) lyase (OASTL) (Shibagaki and Grossman, 2006, 2010). In Arabidopsis, 12 Sultrs have been functionally characterized and divided into four groups according to their phylogenic relationships and kinetic properties (Takahashi et al., 2000; Takahashi, 2019). Group 1 has three members (*AtSultr1;1, AtSultr1;2*, and *AtSultr1;3*), of which *AtSultr1;1* and *AtSultr1;2* encode high-affinity Sultrs found in the epidermis and the cortex of roots, which are mainly responsible for the uptake of ${\text{SO}}_{4}^{2-}$ from the soil (Yoshimoto et al., 2002) and are negatively regulated by cytokinins (Maruyama-Nakashita et al., 2004), while *AtSultr1;3* is localized in the phloem and is involved in the source-to-sink transport of ${\text{SO}}_{4}^{2-}$ (Yoshimoto et al., 2003). The members of Group 2 (*AtSultr2;1* and *AtSultr2;2*) encode low-affinity Sultrs that are expressed in the root phloem and leaf vascular bundle sheath cells and mediate the long-distance transportation of ${\text{SO}}_{4}^{2-}$ from the root to the shoot (Takahashi et al., 2000). Group 3 contains five members that are localized in the chloroplast membrane, which participate in ${\text{SO}}_{4}^{2-}$ uptake by chloroplasts and influence downstream ${\text{SO}}_{4}^{2-}$ assimilation (Cao et al., 2013; Chen et al., 2019). Moreover, the Group 3 Sultr genes may promote stress-induced Cys synthesis, which, in turn, triggers abscisic acid (ABA) biosynthesis to regulate stomatal closure, thereby enhancing plant resistance to abiotic stress (Batool et al., 2018; Chen et al., 2019; Rajab et al., 2019). Furthermore, *AtSultr3;5* has been reported to enhance the root-to-shoot ${\text{SO}}_{4}^{2-}$ transport capacity of *AtSultr2;1* (Kataoka et al., 2004a). Group 4 (*AtSultr4;1* and *AtSultr4;2*) is localized in the vacuolar membrane, influences the efflux and storage of ${\text{SO}}_{4}^{2-}$ to optimize the internal distribution of ${\text{SO}}_{4}^{2-}$ (Kataoka et al., 2004b), and is regulated by ethylene to alleviate S deficiency in oilseed rape (Al Murad et al., 2020).

In addition to Arabidopsis, Sultr genes have been well-characterized in other species. For example, the overexpression of *GmSULTR1;2b* in tobacco showed greater ${\text{SO}}_{4}^{2-}$ transport, allowing the accumulation of more ${\text{SO}}_{4}^{2-}$ and S-containing amino acids, which enhanced its tolerance to drought and S-deficiency stress (Ding et al., 2016). *OsSultr1;1* was functionally similar to *AtSultr1;1*, which encoded a high-affinity transporter specifically expressed in the roots and was induced by S-deficiency. In addition, the overexpression of *OsSultr1;1* in Arabidopsis enhanced its heavy metal stress tolerance under S-deficiency conditions (Kumar et al., 2011, 2019). However, the Group 3 Sultrs in rice and Arabidopsis show different subcellular localizations and functions (Zhao et al., 2016; Yamaji et al., 2017), indicating the species-specific function of Sultrs. To date, previous studies have mainly focused on model plants and field crops, while only a few studies have reported on horticultural crops (Takahashi, 2019). Studies in horticultural crops have been limited to the identification of 12 BSultr members in *Brassica oleracea* (Buchner et al., 2004a), 8 CsSultr genes in tea plants that are involved in the response to selenium (Zhang et al., 2021), and 18 PtaSultrs in poplar trees that regulate the distribution of ${\text{SO}}_{4}^{2-}$ in the phloem at the transcriptional level (Dürr et al., 2010). Although some Sultr members have been identified in horticultural crops, relatively few Sultrs have been functionally characterized, and the basic knowledge of Sultrs in apples has not yet been reported.

Globally, apples are one of the most important economic fruit trees. Sulfur, an essential component of S-rich amino acids and proteins, is indispensable for maintaining the growth and development of fruit trees. However, for a long time, agricultural production has focused on the input of nitrogen, phosphorus, and potassium, neglecting the supplements of S nutrition. This has caused the deficiency of S in soil, which has become a limiting factor for the production and development of fruit trees (Kulczycki, 2021). The uptake of ${\text{SO}}_{4}^{2-}$ from the soil by the roots is an initial and critical step for plants to acquire S, and rootstocks serve as the root system of the cultivated apple tree. *Malus hupehensis* is widely used as an excellent *Malus* rootstock, has apomictic characteristics, and is sensitive to soil nutrient changes (Yang et al., 2008; Yang and Fan, 2012). Therefore, identifying the key genes affecting ${\text{SO}}_{4}^{2-}$ uptake and determining the regulatory mechanisms are necessary to improve the utilization of S in fruit trees.

In this study, nine *MdSultr* genes were identified based on the apple (*Malus domestica*) genome database, and their phylogenetic relationships, gene structure, conserved motifs, cis-acting elements, tissue expression patterns, and expression profiling were analyzed in response to low S and hormones. Notably, *MdSultr3;1a* was especially expressed in the roots and significantly and rapidly responded to low-S stress. Subsequently, *MhSultr3;1a* was isolated from *M. hupehensis* roots, and its function was further characterized by overexpression in yeast and apple calli, laying the foundation for an in-depth analysis of the function of *MdSultrs* and their regulatory mechanism.

## Materials and Methods

### Plant Materials and Treatments

The different *Malus* tissues used for the tissue-specific expression analyses were obtained from 7-year-old “Royal Gala” apple trees at the experimental station of the Shandong Agricultural University in 2020. The roots, stems, and leaves were collected, immediately frozen in liquid nitrogen, and stored at −80°C.

The tissue-cultured “Royal Gala” seedlings used for a low-S and hormone treatment were grown on a Murashige and Skoog (MS) agar medium supplemented with 30 g/L of sucrose and 0.3 mg/L of indole-3-acetic acid (IBA) for 30 days under 16 h light/8 h dark conditions at 26°C. Consistently growing and rooted tissue culture seedlings were selected and transferred into tissue culture glass bottles (250 ml) containing a half-strength Hoagland nutrient solution for treatment. The tissue culture glass bottles were wrapped with black plastic to simulate the dark environment of roots in the soil. The nutrient solution was continuously aerated with an air pump and the dissolved oxygen concentration was maintained at 8–8.5 mg/L. Additionally, the solution was replaced every 2 days. After 7 days of pre-cultivation, the seedlings were prepared for the following treatment: (1) the seedlings were treated in a half-strength Hoagland's nutrient solution (Control, CK) and an half-strength Hoagland's nutrient solution lacking S supplemented with 0.1 mM of MgSO_4_ (low S treatment), and the samples of the roots and leaves were collected at 0, 1, 3, 6, 9, 12, and 15 days after the treatment; (2) for the hormone treatments, the seedlings were treated with a half-strength Hoagland's nutrient solution containing either 5 μM of ABA, 2 μM of indole-3-acetic acid (IAA), or 50 μM of methyl jasmonate (MeJA) for 0, 3, 6, 9, and 12 h and 1, 3, and 7 days before the roots were collected. All of the collected samples were quickly frozen in liquid nitrogen and stored at −80°C for a qRT-PCR analysis.

*Malus hupehensis* seedlings were used for gene cloning. Its seeds were soaked in water for 24 h and then mixed with fine sand at 4°C stratifications for 40 days. The germinated seeds were cultivated in plastic pots filled with soil that was mixed with turf, perlite, and vermiculite (3:1:1) in the greenhouse until they produced eight leaves.

The apple calli (“Orin” cultivar) used for genetic transformation were grown on an MS medium containing 0.5 mg/L of 2,4-dichlorophenoxyacetic acid (2,4-D) and 1.5 mg/L of 6-benzylaminopurine (6-BA) at 26°C in the dark and were sub-cultured in 16-day intervals.

### Identification and Phylogeny of the *MdSultr* Gene Family in *Malus domestica*

To identify *MdSultr* genes in the *Malus domestica* genome, 12 Arabidopsis Sultrs protein sequences were downloaded from The Arabidopsis Information Resource (TAIR) (https://www.arabidopsis.org/) according to the gene accession numbers (Supplementary Table 1) reported previously (Takahashi, 2010) and used as queries to perform local Protein Basic Local Alignment Search Tool (BLASTP) searches in the apple protein database downloaded from Pfam (http://pfam.xfam.org/). Additionally, the Hidden Markov Model (HMM) profiles of the Sulfate_transp (PF00916) and STAS domain (PF01740) were downloaded from the Pfam database and utilized to search for the apple protein database using HMM 3.0 (Harvard University, USA). Afterward, the results were combined and duplicate gene sequences were deleted. Finally, the results were submitted to the Simple Modular Architecture Research Tool (SMART) database (http://smart.embl-heidelberg.de/) and the Conserved Domain Database (CDD) of National Center for Biotechnology Information (NCBI) (https://www.ncbi.nlm.nih.gov/cdd/?term=) to eliminate the genes that did not contain complete Sulfate_transp and STAS domains.

All candidate sequences were submitted to the Expert Protein Analysis System (ExPaSy) Proteomics Server online tools (http://web.expasy.org/protparam/) to predict molecular weight (MW), isoelectric point (PI), the number of amino acids (AAs), and the length of the open reading frame (ORF). The subcellular localization of *MdSultrs* was predicted using Plant-mPLoc (http://www.csbio.sjtu.edu.cn/bioinf/plant-multi/).

Multiple sequence alignments of Sultr protein sequences in apple and Arabidopsis were generated using the multiple sequence comparison by log-expectation (MUSCLE) program (Edgar, 2004) (GenBank accession numbers see in Supplementary Table 1), and the alignment data were used to construct a neighbor-joining (NJ) phylogenetic tree using MEGA 7.0 (King Abdulaziz University, Jeddah, Saudi Arabia) with the following parameters: 1,000 bootstrap replications, a passion model, and pairwise deletion (Kumar et al., 2016).

### Chromosomal Locations, Gene Structure, Conserved Motif, and Promoter Analysis of *MdSultrs*

The chromosomal location information of the *MdSultr* genes was obtained from the Genome Database for Rosaceae (GDR) (http://www.rosaceae.org/) and submitted to MapGene2chrom (MG2C) (http://mg2c.iask.in/mg2c_v2.0/) to draw the chromosome location images. The information about the gene structure of *MdSultrs* was extracted from genome annotation files. The conserved motifs of *MdSultrs* were analyzed by the MEME program Version 5.3.3 (http://meme-suite.org/tools/meme). The region 2,000 bp upstream of the transcription start site of the *MdSultrs* was extracted and then submitted to the PlantCARE software (UGent, Gent, Belgium) (http://bioinformatics.psb.ugent.be/webtools/plantcare/html/) to analyze the cis-acting elements related to stress responsiveness and plant hormones on the promoter regions of *MdSultrs*. Additionally, TBtools (South China Agricultural University, Guangzhou, China) were used for visualization (Chen et al., 2020a).

### Expression Profile Analysis of *MdSultrs* in Different Tissues

To investigate the tissue-specific expression of nine *MdSultrs*, the gene expression profile data of apples were obtained from the National Center for Biotechnology Information (NCBI)-Gene Expression Omnibus (GEO) (https://www.ncbi.nlm.nih.gov/geo/) database (GSE42873). The expression matrix of nine *MdSultrs* from various tissues in different apple cultivars was extracted and visualized by TBtools (Chen et al., 2020a).

### *MhSultr3;1a* Isolation and Sequence Analysis

The coding sequence of *MhSultr3;1a* (GenBank accession No.: MZ634458) was amplified from the cDNA of *M. hupehensis* roots with specific primers, namely, *MhSultr3;1a-F* and *MhSultr3;1a-R* (Supplementary Table 2), using a Phanta Max Super-Fidelity DNA Polymerase Kit (Vazyme, Nanjing, China) according to the instructions of the manufacturer. Then, PCR amplification was performed as follows: 5 min at 95°C, 35 cycles at 94°C for 30 s, 56°C for 30 s, 72°C for 2 min, and a final extension at 72°C for 10 min. The PCR products were checked by sequencing (BGI, Shenzhen, China).

Multiple sequence alignments were performed using DNAman (Lynnon Biosoft, San Ramon, CA, United States). The full-length amino acid sequences of Sultr3;1s from *M. domestica, Pyrus bretschneideri, Prunus avium, Prunus persica, Populus trichocarpa*, and Arabidopsis (GenBank accession numbers see in Supplementary Table 1) were used for phylogenetic analysis with MEGA7.0 (Kumar et al., 2016).

### Subcellular Localization of *MhSultr3;1a*

The coding sequence of *MhSultr3;1a* was amplified with the specific primers: *MhSultr3;1a-EF* and *MhSultr3;1a-ER* (Supplementary Table 2). The amplified fragment was first cloned into the entry vector pDONR222 and subsequently into the binary vector pGWB405-GFP, which are controlled by the CaMV *35S* promoter using the Gateway BP and LR recombination reactions, respectively (Invitrogen, Carlsbad, CA, USA). For subcellular localization, *Agrobacterium* GV3101 containing the *35S:MhSultr3;1a-GFP* fusion vector or empty vector *35S: GFP* was inserted into the leaf epidermal cells of *Nicotiana benthamiana* (Chen et al., 2018). The infiltrated tobacco was cultured in the dark for 48 h, and afterward, the infected leaves were cut and observed with a high-resolution laser confocal microscope (ZEISS LSM 880, Jena, Germany).

### Yeast Complementation and Growth Analysis of *MhSultr3;1a*

To construct the plasmid p112AINE-MhSultr3;1a, the coding sequence of *MhSultr3;1a* was amplified with specific primers, namely, *MhSultr3;1aYF* and *MhSultr3;1aYR* (Supplementary Table 2), and then inserted into the EcoRI and BamHI sites of the p112AINE yeast expression vector using the ClonExpress II One Step Cloning Kit (Vazyme, Nanjing, China) (Ding et al., 2016). The recombinant vector MhSultr3;1a-p112AINE and the empty vector p112AINE were transferred into the yeast double ${\text{SO}}_{4}^{2-}$ transporter mutant CP154-7A (*MATa, his3, leu2, ura3, ade2, trp1, sul1::LEU2*, and *sul2::URA3*) using the lithium acetate method and then selected on a synthetic defined (SD) yeast medium without Tryptophan at 28°C over 3 days (Cherest et al., 1997). For the complementation assays, the transformed yeast was cultured in a liquid SD/-Trp medium until OD_600_ =1 and diluted to four sequential dilutions: 10^−1^, 10^−2^, 10^−3^, and 10^−4^; subsequently, 20 μl of each dilution were dropped onto plates with a yeast nitrogen base (YNB) medium (without ammonium ${\text{SO}}_{4}^{2-}$), which was supplemented with essential amino acids and 0.1 mM of ${\text{SO}}_{4}^{2-}$ or 0.1 mM of homocysteine as the sole S source. All plates were incubated at 28°C for 3 days to observe growth.

To measure the growth rate of the yeast, the CP154-7A, p112AINE/CP154-7A, and MhSultr3;1a-p112AINE yeasts were incubated in a YNB liquid medium (without ammonium ${\text{SO}}_{4}^{2-}$) that was supplemented with essential amino acids and 0.1 mM of sodium ${\text{SO}}_{4}^{2-}$. The OD_600_ values were measured every 6 h for 84 h using a microplate spectrophotometer (Fisher Scientific, Hampton, NH, USA) with three replicates.

### Apple Calli Transformation and Treatment

The resulting vector, *MhSultr3;1a*-GFP, was transformed into *Agrobacterium* strain LBA4404 using the heat shock method. The transgenic apple calli were generated as described previously (An et al., 2017). Additionally, the transgenic apple calli were confirmed by PCR at DNA and RNA levels (Supplementary Figure 1). A Plant Genomic DNA Kit (Beijing Bioteke Corporation) was used to extract the DNA. The primers used for PCR are listed in Supplementary Table 3.

The 15-day-old wild type (WT) and the transgenic apple calli (OE1 and OE2) were transferred to an MS agar medium (CK) or MS agar medium (without S) containing 0.1 mm of MgSO_4_ (low S) and then photographed after 15 days. The fresh weight, ${\text{SO}}_{4}^{2-}$ content, and Cys content were measured. The Cys content was measured using a Cys assay kit (Solarbio Science & Technology, Beijing, China). The ${\text{SO}}_{4}^{2-}$ content was determined as described previously (Lancilli et al., 2014). The apple calli grown on each plate were used as one biological replicate. A total of three biological replicates were analyzed. For each biological replicate, ${\text{SO}}_{4}^{2-}$ content, and Cys content were determined using 0.1 g of calli, respectively.

### Total RNA Extraction and qRT-PCR Analysis

Total RNA was extracted from “Royal Gala,” *M. hupehensis*, and apple calli using the polysaccharides- and polyphenolics-rich RNAprep Pure Plant Kit (Tiangen Biotech, Beijing, China). Then, 1 μg of the total RNA was used to synthesize the cDNA using the PrimeScript™ RT reagent Kit and gDNA Eraser (perfect real-time) according to the instructions of the manufacturer (Takara Bio Inc., Shiga, Japan). Afterward, qRT-PCR was performed on a LightCycler^®^96 (Roche) using the TB Green^®^ Premix Ex Taq™ (Tli RNaseH Plus) (TaKaRa, Japan) under the following conditions: 45 cycles of 95°C for 5 s and 60°C for 30 s. All primers used for the qRT-PCR are shown in Supplementary Table 4. Mdactin was used as the reference gene. Three biological and technical replications were assayed and the data were calculated using the 2^−ΔΔCt^ method.

### Statistical Analyses

Statistical analyses were performed with the Student's *t*-test and one-way ANOVA tests using the DPS 7.05 software (Shenzhen Chinese Technology Co., Ltd). All experiments were carried out in triplicates and expressed as the mean ± SD. The confidence level for statistical significance was *p* < 0.05. The graphs were made using Microsoft Excel 2010 (Microsoft Corp., Redmond, WA).

## Results

### Identification and Chromosomal Location of *MdSultrs*

**Figure 1:**
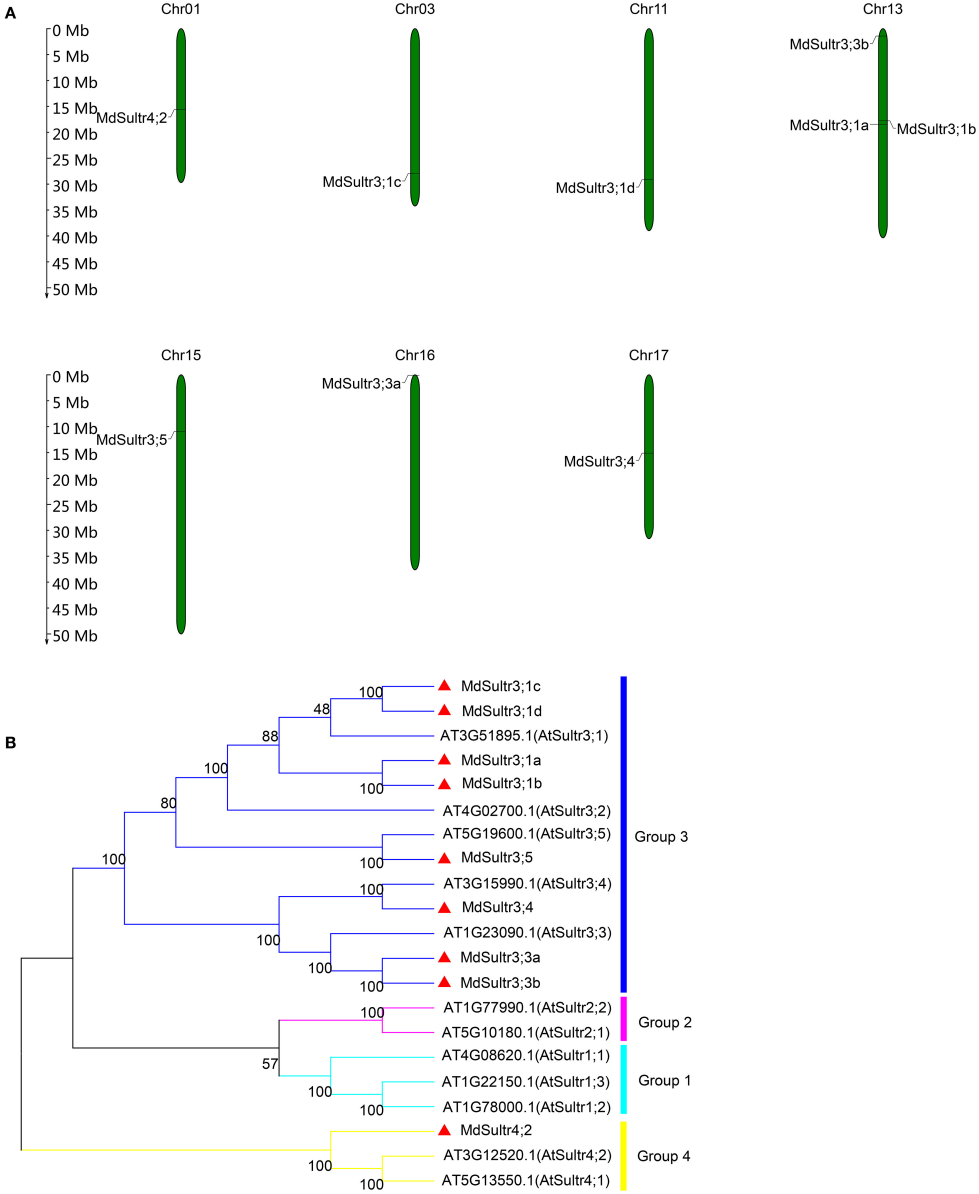
Chromosome location of *MdSultrs* and phylogenetic analysis of *Malus domestica* and Arabidopsis sulfate transporters (Sultr) proteins. **(A)** Genomic locations of nine *MdSultrs* on *M. domestica* chromosomes. **(B)** The phylogenetic analysis of 12 *AtSultrs* and 9 *MdSultrs* using the MEGA 7.0 software using the neighbor-joining (NJ) method with 1,000 bootstrap values using protein sequences. The *MdSultrs* were divided into two groups (red triangle). The different groups were displayed in different colors.

To identify *MdSultrs* members, the protein sequences of 12 *AtSultrs*, the conserved Sulfate_transp, and STAS domains were used as queries to search the *M. domestica* genome database using the BLASTP program. Afterward, the results were combined and the duplicate gene sequences were deleted. The genes that did not contain the complete Sulfate_transp and STAS domains were removed using SMART and CDD. Finally, nine putative *MdSultrs* were identified. As shown in Table 1, the ORF length of the *MdSultrs* ranged from 1,866 to 4,878 bp, encoding 622–1,625 AAs. *MdSultr3;5* had the longest ORF and the most AAs. The predicted MW of the MdSultrs was between 68.2 and 181.1 kDa. The theoretical pI values ranged from 5.52 to 9.04. The subcellular localization prediction results show that *MdSultr3;5* was predicted to localize to the nucleus, while *MdSultr4;2* was predicted to localize to the chloroplast. The remaining seven *MdSultrs* were predicted to localize to the cellular membrane. According to the genomic location information obtained from the apple genome database, the nine *MdSultrs* were mapped onto apple chromosomes (Chrs) 1, 3, 11, 13, 15, 16, and 17. Among them, Chr13 contained the most *MdSultr* genes (three), whereas the other Chrs contained only one *MdSultr* gene each (Figure 1A).

**Table 1:** Genes and proteins characteristic information of *MdSultrs*.

**Gene name**	**Gene ID**	**Genomic location**	**ORF**	**Number of amino acids(AAs)**	**pI**	**MW(kDa)**	**Subcellular location**	**Percent identity (%)**
MdSultr3;1a	MDP0000085223	chr13:20276286.0.20281261	1,866	622	8.74	68.2	Cell membrane	70.23
MdSultr3;1b	MDP0000216466	chr13:19601922.0.19606653	1,977	658	8.75	72.0	Cell membrane	70.37
MdSultr3;1c	MDP0000231619	chr3:30903274.0.30909128	1,914	637	7.61	70.3	Cell membrane	73.85
MdSultr3;1d	MDP0000311618	chr11:32015041.0.32020795	1,974	657	8.59	72.3	Cell membrane	75.91
MdSultr3;3a	MDP0000145668	chr16:115524.0.120325	2,112	703	8.88	77.4	Cell membrane	70.11
MdSultr3;3b	MDP0000190006	chr13:1507311.0.1512218	2,184	727	9.04	79.9	Cell membrane	68.86
MdSultr3;4	MDP0000317974	chr17:16675271.0.16680286	1,992	663	8.67	72.5	Cell membrane	73.16
MdSultr3;5	MDP0000167489	chr15:12003012.0.12018806	4,878	1,625	5.52	181.1	Nucleus	65.51
MdSultr4;2	MDP0000141450	chr1:17194702.0.17202334	2,175	724	6.81	79.6	Chloroplast	65.55

### Phylogenetic and Structural Characterization Analysis of *MdSultrs*

**Figure 2:**
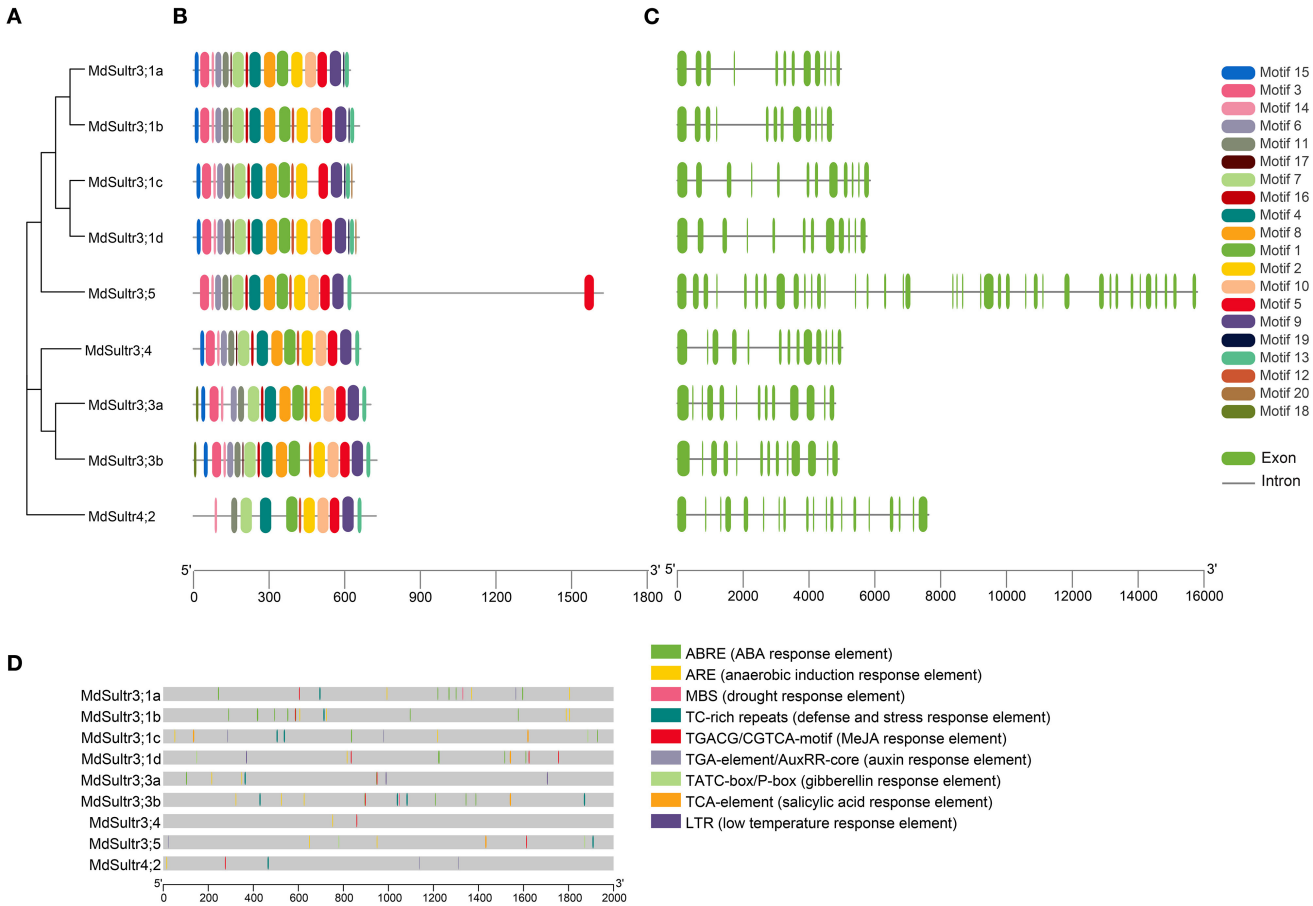
Phylogenetic relationship, conserved motifs, gene structures, and promoter analysis of *MdSultrs*. **(A)** Phylogenetic analysis of nine *MdSultr* proteins. The phylogenetic tree was constructed based on the full-length protein sequences of nine *MdSultr* proteins using MEGA 7.0. **(B)** Distributions of conserved motifs in *MdSultr* proteins. The motifs are displayed in different colors. For the details of the motifs, refer to Supplementary Table 5. **(C)** The exon-intron structure of *MdSultr* genes. The green boxes represent exons and the gray lines represent introns. **(D)** The promoter analysis of *MdSultrs*. The potential cis-elements in the promoter regions 2,000 bp upstream of the *MdSultrs* were analyzed by PlantCare software, and the elements related to stress responsiveness and plant hormones were shown. Different color boxes indicate different cis-acting elements.

To understand the phylogenetic relationship between *M. domestica* and Arabidopsis, an NJ-phylogenetic tree was constructed based on the protein sequence alignment of the full sequences of the 9 *MdSultrs* and 12 *AtSultrs* proteins. As shown in Figures 1B, 2A, the nine putative MdSultrs were divided into two groups, with Group 3 included eight members (*MdSultr3;1a, MdSultr3;1b, MdSultr3;1c, MdSultr3;1d, MdSultr3;3a, MdSultr3;3b, MdSultr3;4*, and *MdSultr3;5*), which had 66–76% similarity with Arabidopsis, and the remaining member (*MdSultr4;2*) was classified as Group 4, which had 66% similarity with Arabidopsis (Table 1).

As shown in Figure 2B, 18 putative conserved motifs with 8~50 amino acid residues were identified using the online MEME tool and subsequently annotated with Pfam and SMART (detailed in Supplementary Table 5). Motifs 5 and 9, which were STAS domains, and Motifs 1, 2, 4, 7, and 11, which were Sulfate_transp domains, were identified in all *MdSultr* proteins, and the remaining motifs were unknown functional elements. Notably, Motif 18 was only identified in the *MdSultr3;3a* and *MdSultr3;3b* proteins. Nine *MdSultr* protein sequences had highly conserved motifs and conserved motif orders. The similar motif distribution of MdSultrs indicated that they have a close evolutionary relationship and similar functions.

To identify the gene structure differences between *MdSultrs*, the gene and protein structure of *MdSultrs* were visualized using TBtools, and the results were consistent with the phylogenetic tree analysis. *MdSultr3;5* and *MdSultr4;2* had 39 and 19 exons, respectively, while the other *MdSultrs* had 12–13 each, and all members contained introns (Figure 2C).

### *Cis*-Elements Analysis of *MdSultrs*

To understand the transcriptional regulation mechanisms of the *MdSultrs*, the 2,000-bp promoter regions of the *MdSultrs* were isolated for the analysis of the potential cis-elements using the Plant-CARE database. As shown in Figure 2D, all *MdSultrs* possessed the anaerobic induction response element (ARE). Except for *MdSultr3;1c*, the other *MdSultrs* contained the MeJA response element (TGACG-motif/CGTCA-motif). Five *MdSultrs* had an ABA response element (ABRE) except for *MdSultr3;4, MdSultr3;5*, and *MdSultr4;2*. *MdSultr3;1a, MdSultr3;1c, MdSultr3;5*, and *MdSultr4;2* had an auxin response element (TGA-element/AuxRR-core). Moreover, hormone-responsive elements, including gibberellin response (TATC-box/P-box) and salicylic acid (SA) response (TCA-element), and stress elements, such as defense and stress (TCA-rich repeats), low temperature (LTR), and drought (MBS) response elements, were found in the *MdSultr* promoter regions. These results suggest that *MdSultrs* may play important roles in the response to hormones and various stressors.

### Expression Pattern of *MdSultrs* in Different Tissues

**Figure 3:**
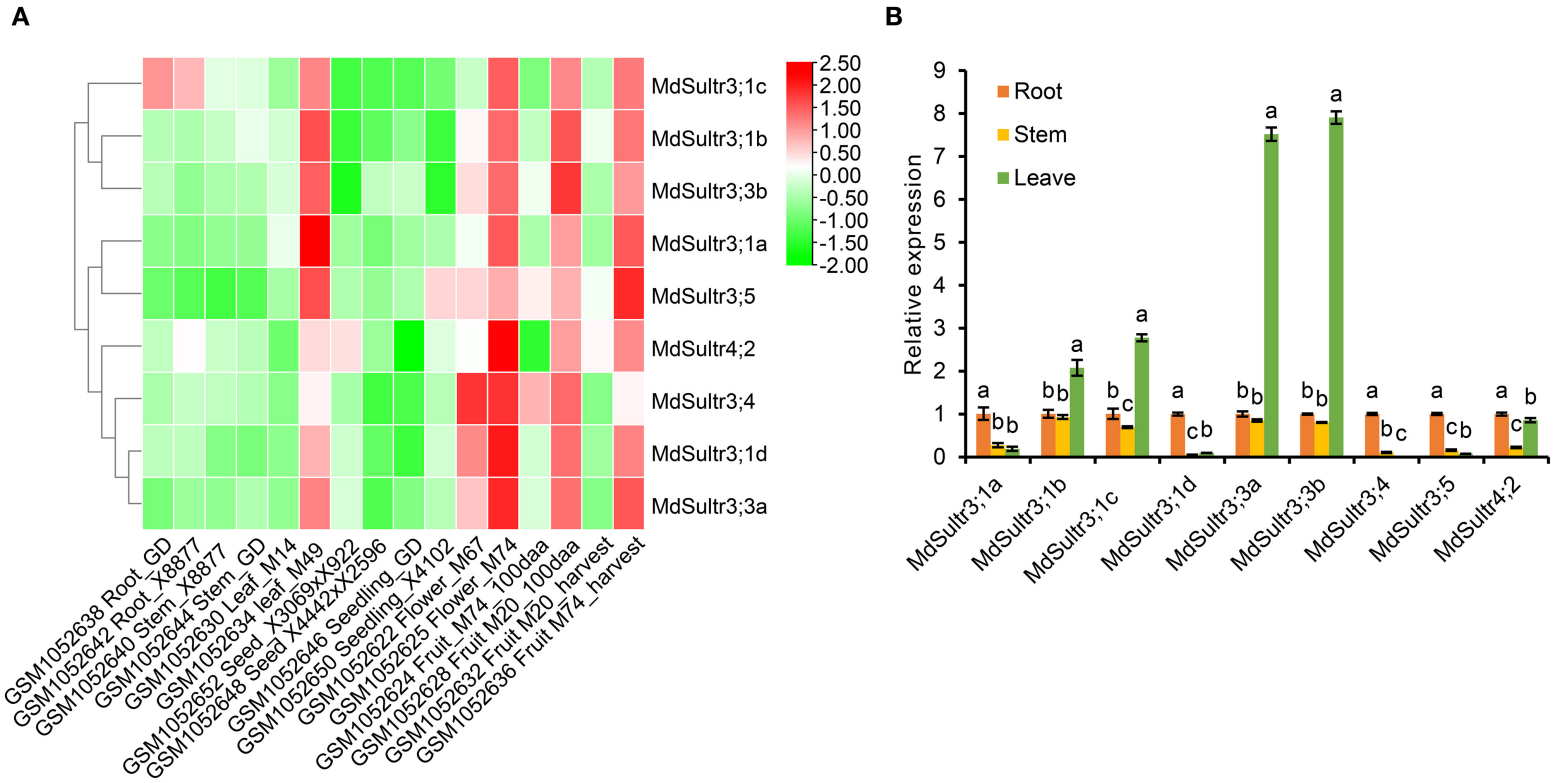
Expression profiles of *MdSultrs* in different tissues of *M. domestica*. **(A)** NCBI-GEO data (GSE42873) were downloaded for the expression profile analysis, and the heatmap was constructed using TBtools. GSM1052638 and GSM1052642 represent the root of Golden Delicious (GD) and X8877, respectively. GSM1052640 and GSM1052644 represent the fully developed stem of X8877 and GD, respectively. GSM1052630 and GSM1052634 represent the whole leaf of M14 and M49, respectively. GSM1052652 represents the dormant seed from cross X3069 and X922. GSM1052648 represents the dormant seed from cross X4442 and X2596. GSM1052646 and GSM1052650 represent the 10-day-old seedlings of GD and X4102, respectively. GSM1052622 and GSM1052625 represent the whole flower of M67 and M74, respectively. GSM1052624 and GSM1052628 Fruit_100aa represents represent the 100 days after anthesis-fruit of M74 and M20, respectively. GSM1052632 and GSM1052636 represent fruit flesh at harvest of M20 and M74, respectively. M67, M74, M20, M14, M49, X8877, Golden Delicious (GD), X41002, X4442, and X2596 represent apple cultivars. **(B)** The expression levels of *MdSultrs* in the root, stem, and leaf of “Royal Gala” using qRT-PCR analysis. Data are presented as the mean ± SD of three independent biological replicates. Different letters indicate significant differences (*p* < 0.05).

To explore the potential function of *MdSultrs* in *M. domestica*, the expression patterns of nine *MdSultrs* in different tissues, including flowers, fruits, leaves, roots, stems, seeds, and seedlings were analyzed using the GEO database (GSE42873). The results showed that nine *MdSultrs* was mainly expressed in the leaves, flowers, and fruits of different apple cultivars and hybrids. *MdSultr3;1c* showed higher expression levels compared with the other eight genes, in the roots of apple cultivars, namely, Golden Delicious (GD) and X8877 (Figure 3A).

In addition, the expression of nine *MdSultrs* in the roots, stems, and leaves of “Royal Gala” was determined by qRT-PCR. As shown in Figure 3B, *MdSultr3;1a, MdSultr3;1d, MdSultr3;4, MdSultr3;5*, and *MdSultr4;2* had the highest expression levels in the roots;, while *MdSultr3;1b, MdSultr3;1c, MdSultr3;3a*, and *MdSultr3;3b* had the highest expression levels in leaves; and *MdSultr4;2* was at relatively high levels in the roots and leaves. In summary, the different expression patterns of *MdSultrs* suggest that *MdSultrs* may play different roles in the growth and development of *Malus*.

### Expression of *MdSultrs* in Response to Low S and Plant Hormones

**Figure 4:**
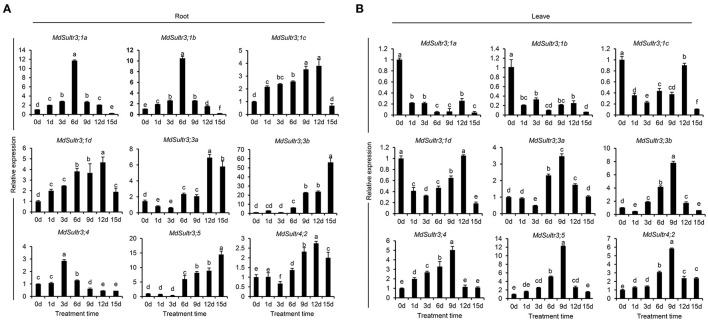
Expression analysis of the *MdSultrs* in the roots **(A)** and leaves **(B)** of “Royal Gala” under low S (0.1 mM of MgSO_4_) condition. Data are presented as the mean ± SD of three independent biological replicates. Different letters indicate significant differences (*p* < 0.05).

To estimate the response of *MdSultrs* to low S, qRT-PCR was used to detect the expression levels of *MdSultrs* in the roots and leaves within 15 days of a low-S treatment. As shown in Figure 4, the expression patterns of *MdSultrs* in the roots and leaves were different under a low-S treatment. For example, in the roots, the expression of *MdSultr3;1a, MdSultr3;1b, MdSultr3;1c*, and *MdSultr3;1d* were significantly and rapidly increased, while *MdSultr3;3a, MdSultr3;3b, MdSultr3;5*, and *MdSultr4;2* were upregulated after a long-term (12–15 d) low-S treatment (Figure 4A). In leaves, *MdSultr3;1a, MdSultr3;1b, MdSultr3;1c*, and *MdSultr 3;1d* were significantly downregulated, while the expression of *MdSultr3;3a* and *MdSultr3;3b* significantly increased after 3 days of a low-S treatment. *MdSultr3;4, MdSultr3;5*, and *MdSultr4;2* were rapidly upregulated after 1–3 days of low-S conditions (Figure 4B).

**Figure 5:**
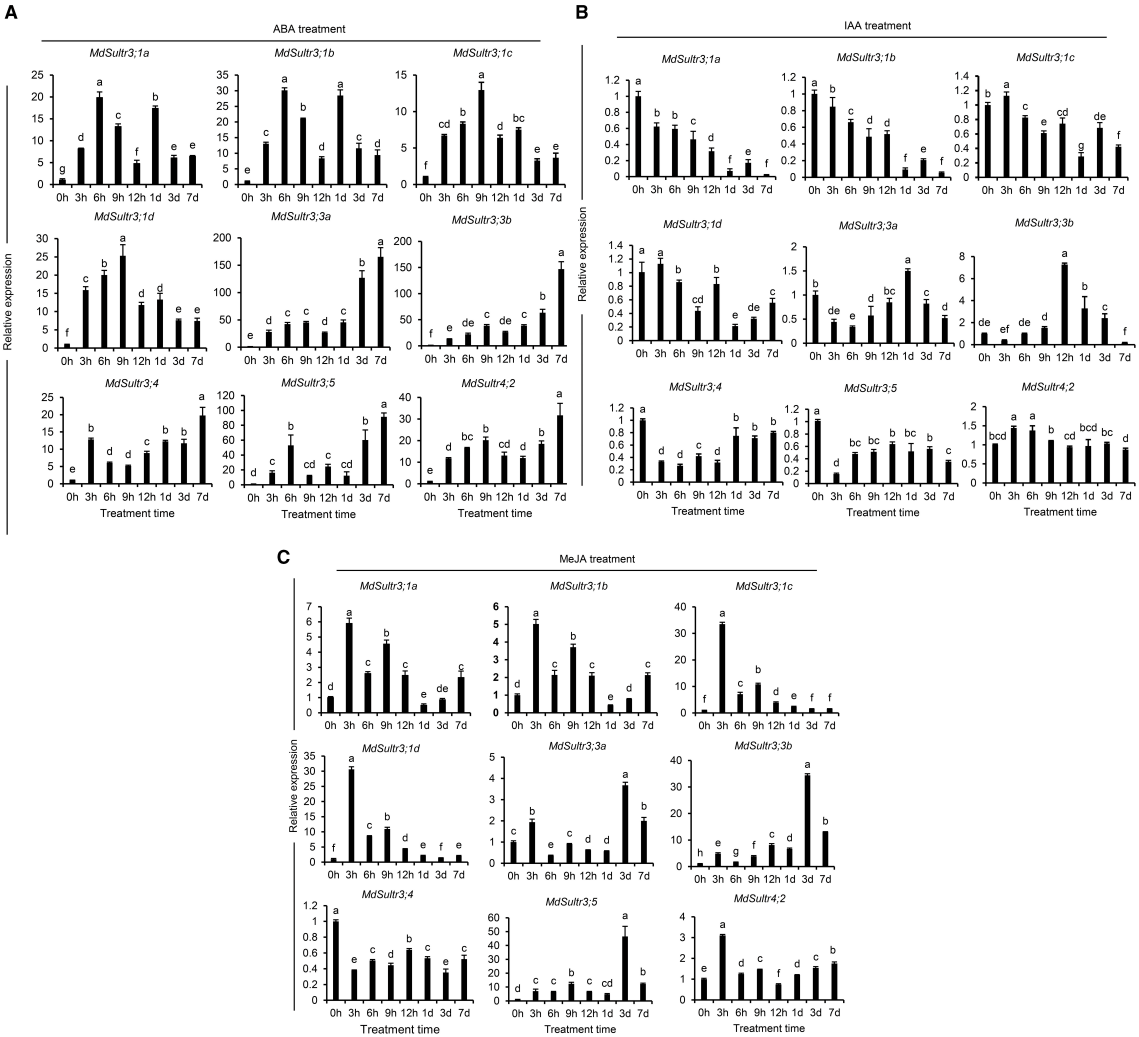
Expression analysis of the *MdSultrs* in the root response to plant hormones. The expression levels of the *MdSultr* genes under 5 μM of abscisic acid (ABA) **(A)**, 2 μM of indole-3-acetic acid (IAA) **(B)**, and 50 μM of methyl jasmonate (MeJA) **(C)** treatments using qRT-PCR analysis. Data are presented as the mean ± SD of three independent biological replicates. The different letters indicate significant differences (*p* < 0.05).

To explore the potential roles of *MdSultrs* in responding to the hormones in roots, the expression patterns of *MdSultrs* were investigated under various plant hormones. As shown in Figure 5, the expression of the nine *MdSultrs* was found to be highly upregulated in response to ABA (Figure 5A). Except for *MdSultr3;3a* and *MdSultr4;2*, which were significantly upregulated by IAA, the other *MdSultr*s were downregulated (Figure 5B). Most *MdSultrs* were upregulated by the exogenous MeJA except for *MdSultr3;4* (Figure 5C). These results indicate that *MdSultrs* may be involved in the crosstalk of hormone signaling with S metabolism.

### Overexpression of *MhSultr3;1a* Improved the Growth of Yeast and Apple Calli Under Low-S Conditions

**Figure 6:**
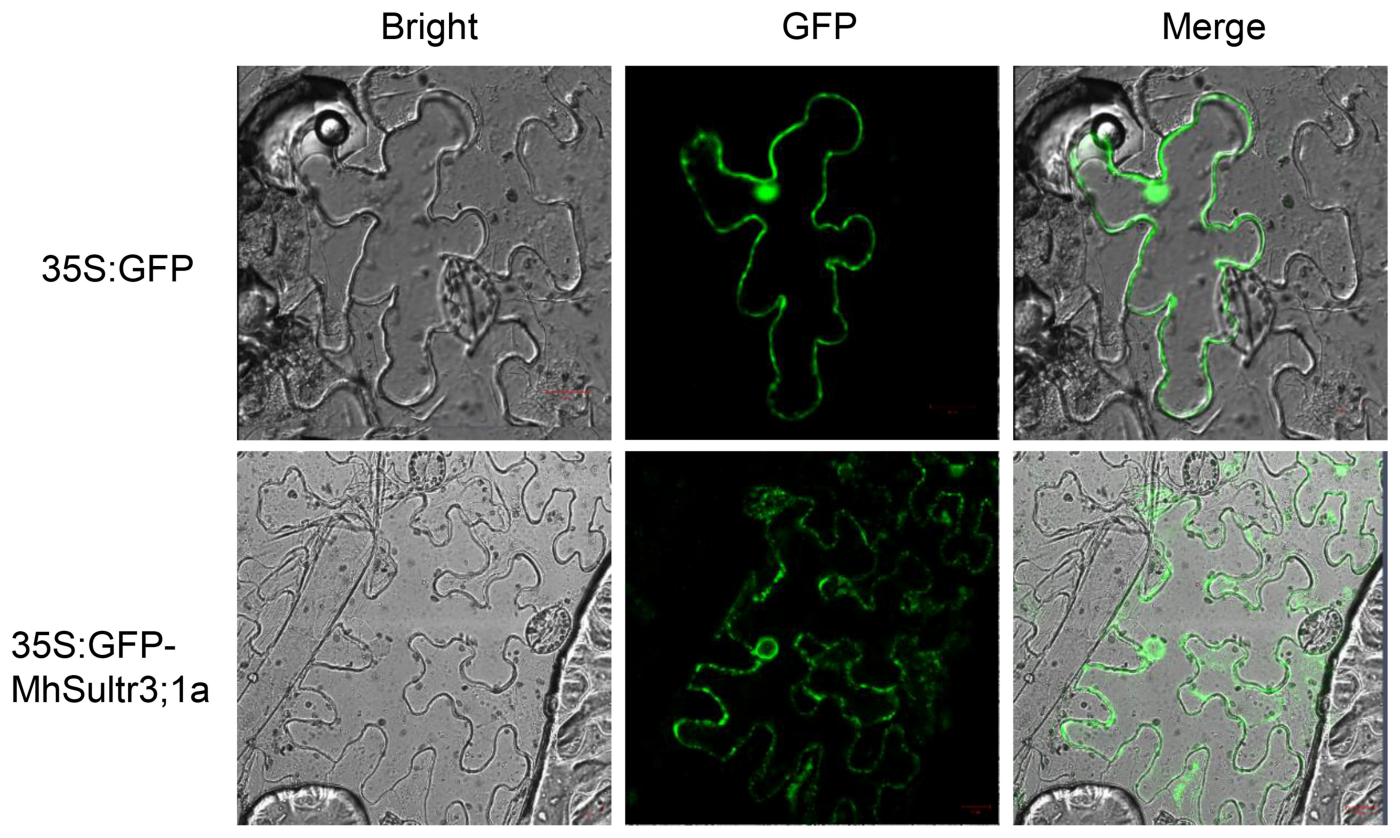
Subcellular localization of the *MhSultr3;1a* protein. *Agrobacterium* GV3101 containing recombinant construct *35S:GFP-MhSultr3;1a* and empty vector *35S:GFP* were inserted into the leaf epidermal cells of *Nicotiana benthamiana*. The fluorescent signals were visualized with a high-resolution laser confocal microscope. The scale bar represents 20 μM.

Considering that *MdSultr3;1a* was especially expressed in roots and induced by low S (Figures 3B, 4), and that previous studies have shown that the genes cloned from *M. hupehensis* are highly homologous to the sequence of the *M. domestica* genome (Liu et al., 2012; Sun et al., 2017; Zhang et al., 2020), *MhSultr3;1a* was isolated from the *M. hupehensis* roots. Multiple alignment showed that the *MhSultr3;1a* protein was highly homologous to the *MdSultr3;1a* from apples (99.85% identity), PbSultr3;1 from *P. bretschneideri* (98.31%), PaSultr3;1 from *P. avium* (92.60%), PpSultr3;1 from *Prunus persica* (92.45%), PtSultr3;1 from *P. trichocarpa* (82.18%), and AtSultr3;1 from Arabidopsis (70.23%) (Supplementary Figure 2A). The phylogenetic tree showed that *MhSultr3;1a* was homologous to several Sultrs, but was most closely related to *MdSultr3;1a* (Supplementary Figure 2B). Subcellular localization revealed that *MhSultr3;1a* was localized to the cell membrane and the nuclear membrane (Figure 6).

**Figure 7:**
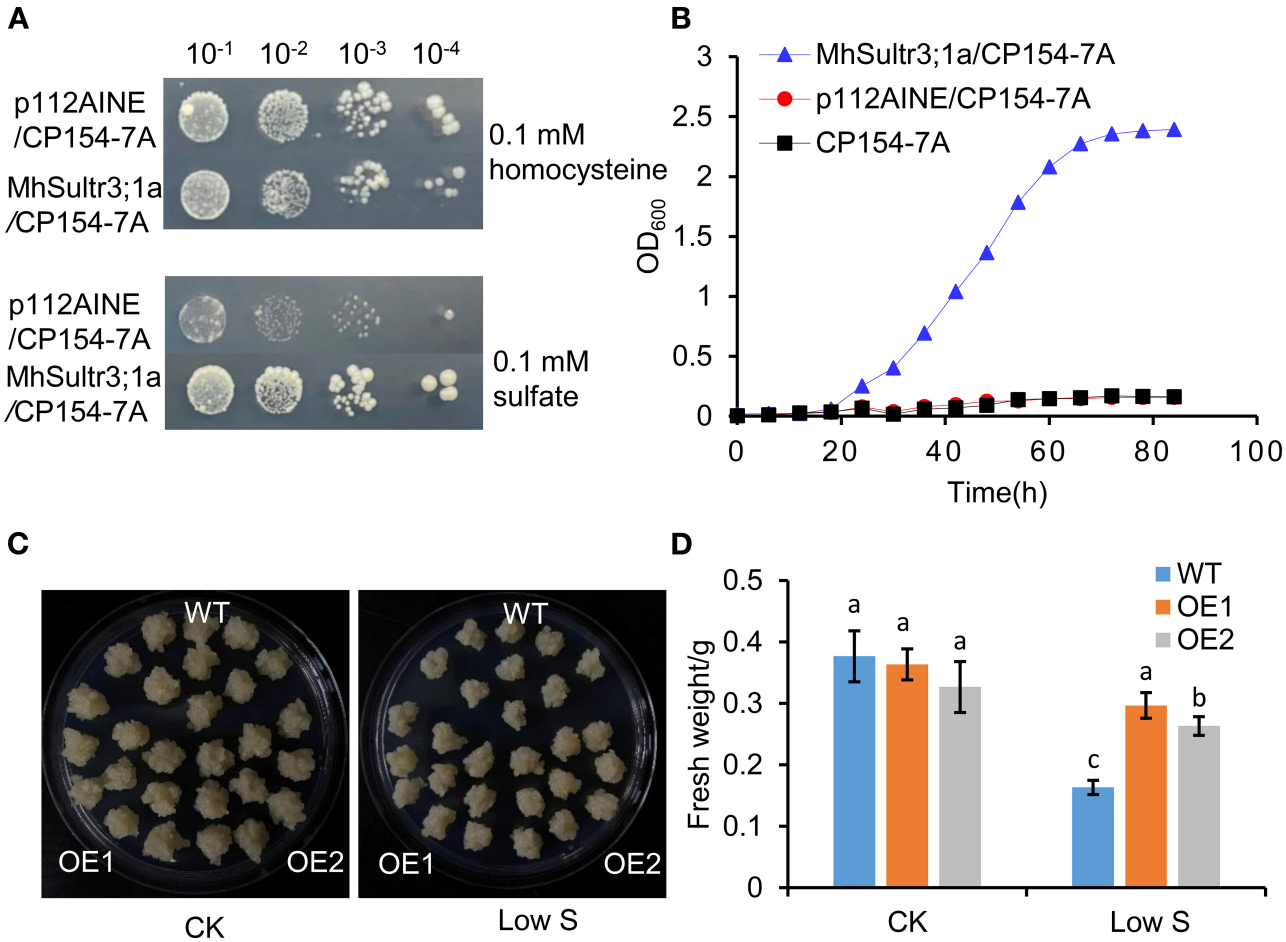
Effects of overexpression of *MhSultr3;1a* on the growth of yeasts and apple calli. **(A)** Complementation of yeast mutant CP154-7A by *MhSultr3;1a*. Yeast cells expressing *MhSultr3;1a* or the empty vector p112AINE were grown at 28°C in liquid SD/-Trp medium until OD_600_ = 1, 20 μl of 10-fold serial dilution was spotted on the YNB plates containing essential amino acids and 0.1 mM of homocysteine or 0.1 mM of sulfate (${\text{SO}}_{4}^{2-}$) as a sole source of sulfur (S). The plates were incubated at 28°C for 3 days. p112A1NE/CP154-7A: CP154-7A mutant transformed with p112A1NE empty vector; MhSultr3;1a/ CP154-7A: CP154-7A mutant transformed with *MhSultr3;1a*. **(B)** The growth curves of yeasts in liquid YNB medium containing essential amino and 0.1 mM of ${\text{SO}}_{4}^{2-}$ as a sole source of sulfur. **(C)** Phenotype comparisons of apple calli between the wild type (WT) and *MhSultr3;1a*-overexpressing transgenic lines (OE1 and OE2) and their **(D)** fresh weight under normal conditions and low-S stress. Data are presented as the mean ± SD of three independent biological replicates. The different letters indicate significant differences (*p* < 0.05).

To investigate whether *MhSultr3;1a* had an ${\text{SO}}_{4}^{2-}$ transport function, complementation tests were performed in the yeast mutant CP154-7A lacking both SUL1 and SUL2 ${\text{SO}}_{4}^{2-}$ transporters. The yeast mutant transformed with *MhSultr3;1a* and the empty vector p112A1NE grew well with 0.1 mM of homocysteine, but only the yeast-transformed *MhSultr3;1a* grew well on a medium containing 0.1 mM of sodium ${\text{SO}}_{4}^{2-}$ as the sole S source (Figure 7A). In contrast, the CP154-7A-transformed p112A1NE did not grow well, indicating that *MhSultr3;1a* can rescue the growth defect of CP154-7A. The growth curve was further analyzed to extend the results, as shown in Figure 7B. The CP154-7A-transformed *MhSultr3;1a* grew well on a liquid YNB medium containing 0.1 mM of ${\text{SO}}_{4}^{2-}$, whereas the CP154-7A- and CP154-7A-transformed p112A1NE failed to grow. Thus, *MhSultr3;1a* complemented the yeast CP154-7A mutant and encoded a functional ${\text{SO}}_{4}^{2-}$ transporter.

To further characterize the function of *MhSultr3;1a*, two lines of transgenic apple calli (OE1 and OE2) were obtained. As shown in Supplementary Figure 1B, OE1 and OE2 showed relatively higher expression levels compared with the WT. No growth differences were observed between the OE1 and OE2 transgenic and WT apple calli under normal conditions, and their growth was inhibited by low-S treatment. Notably, the OE1 and OE2 transgenic apple calli had larger sizes than the WT when transferred to low S supply conditions (Figure 7C), and the fresh weights of OE1 and OE2 were higher than those of the WT (Figure 7D). The overexpression of *MhSultr3;1a* improved the growth of yeast and apple calli under low-S conditions.

### Overexpression of *MhSultr3;1a* Increased the Content of ${\text{SO}}_{4}^{2–}$ and Cys Under Low S Conditions

**Figure 8:**
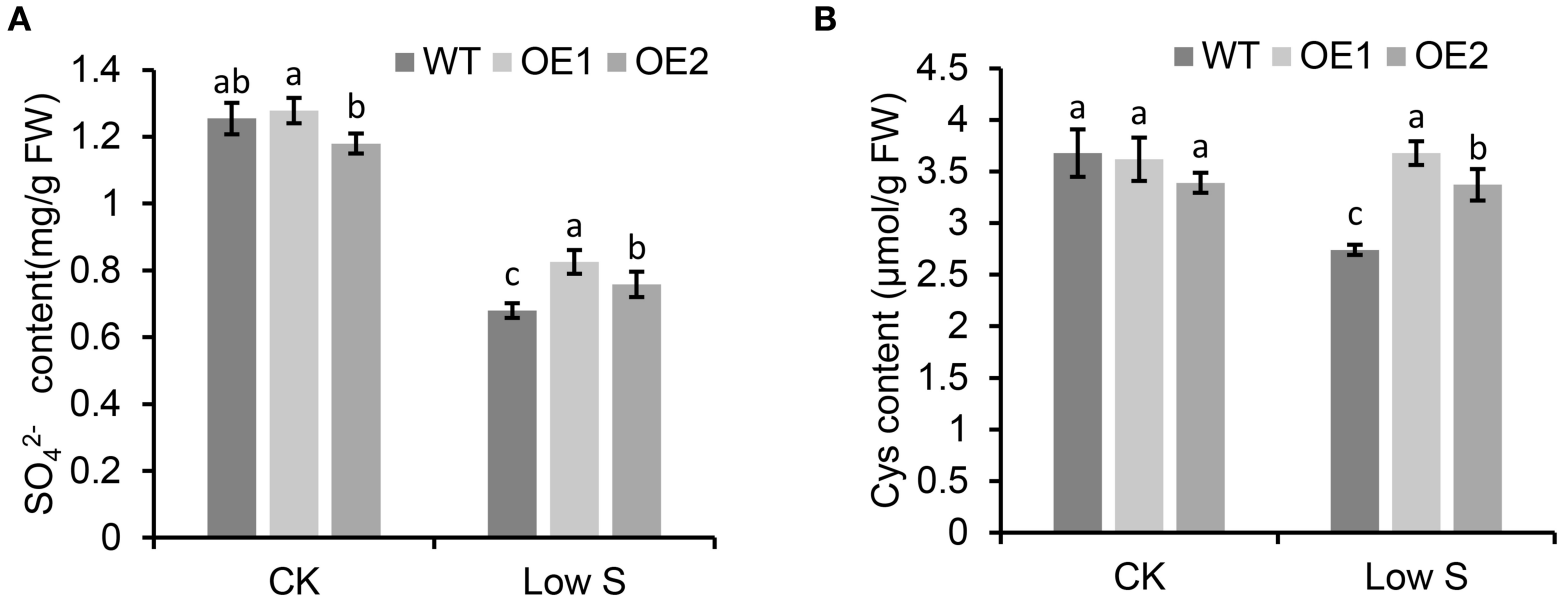
*MhSultr3;1a* enhanced the increased content of ${\text{SO}}_{4}^{2-}$ and cysteine (Cys) under low-S conditions. **(A)** ${\text{SO}}_{4}^{2-}$ and Cys **(B)** content of in transgenic (OE1 and OE2) and WT apple calli under low-S conditions. Data are presented as the mean ± SD of three independent biological replicates. The different letters indicate significant differences (*p* < 0.05).

As shown in Figure 8, the contents of ${\text{SO}}_{4}^{2-}$ (Figure 8A) and Cys (Figure 8B) under normal conditions showed no significant differences in the transgenic apple calli overexpressing *MhSultr3;1a* (OE1 and OE2) and the WT, however, under low-S conditions, the contents of ${\text{SO}}_{4}^{2-}$ and Cys in OE1 and OE2 were significantly higher than those in the WT. Especially in OE1, the Cys content was 34.2% higher than that in the WT under low-S conditions. Similarly, the Cys content in OE2 was also higher than that in the WT by 23.06% (Figure 8B). The overexpression of *MhSultr3;1a* accumulated more ${\text{SO}}_{4}^{2-}$ and Cys contents to meet the demands of S-containing compounds under S-limiting conditions.

## Discussion

Sulfate transporters are encoded by multiple gene families (Gigolashvili and Kopriva, 2014), and 8–28 gene members have been identified in Arabidopsis, rice, maize, and soybeans (Takahashi et al., 2000; Kumar et al., 2011; Ding et al., 2016; Huang et al., 2018). In this study, a total of nine *MdSultr* genes were identified in *M. domestica*, all of which were structurally characterized by the presence of a highly conserved Sulfate_transp domain and STAS domains (Figures 2B,C and Supplementary Table 5), as described in other species such as sorghum, potatoes, and tea trees (Vatansever et al., 2016; Akbudak et al., 2018; Zhang et al., 2021), indicating that *MdSultrs* have the typical characteristics of Sultrs. In addition, the phylogenetic tree analysis separated them into two distinct groups, such that *MdSultr4;2* and the remaining *MdSultrs* were on Group 4 and 3, respectively (Figure 1B). However, the Sultrs of maize (Huang et al., 2018), sorghum (Akbudak et al., 2018), rice (Kumar et al., 2011), and poplar (Dürr et al., 2010) are generally divided into four subfamilies. About 8 *CsSultrs* in the tea plant are also classified into four groups (Zhang et al., 2021). Interestingly, studies in tea plants, corn, and wheat have shown that some genes in Group 1 and 3 are highly homologous and similar in function (Buchner et al., 2010; Huang et al., 2018; Zhang et al., 2021). For example, *PtaSultr3;3a* and *PtaSultr1;1* in poplar are responsible for ${\text{SO}}_{4}^{2-}$ loading into the phloem (Dürr et al., 2010), and *CsSultr1;1, CsSultr1;2*, and *CsSultr 3;2* in tea plants were significantly induced by an exogenous S treatment (Zhang et al., 2021). Similarly, *ZmSultr1;1, ZmSultr1;2*, and *ZmSultr3;4* were upregulated in S-deficient maize roots (Huang et al., 2018). The *MdSultrs* in *M. domestica* were divided into only two groups (Figure 1B), and there were no genes classified as Group 1, indicating that *MdSultrs* may not have excessive functional redundancy compared with other crops.

The expression of *MdSultrs* showed tissue-specific patterns and responded to various hormones. *MdSultr3;1a, MdSultr3;1d, MdSultr3;4*, and *MdSultr3;5* showed significantly higher expression levels in roots than in leaves and stems, while *MdSultr3;1b, MdSultr3;1c, MdSultr3;3a*, and *MdSultr3;3b* were strongly expressed in leaves (Figure 3B). Similarly, their corresponding orthologs, *AtSultr3;1* and *AtSultr3;2* in Arabidopsis, are also specifically expressed in the leaves (Takahashi et al., 2000). The tissue-specific expression patterns of *MdSultrs* indicate that they may play different roles in different tissues. Moreover, *MdSultrs* also responded to multiple hormones. For instance, all *MdSultrs* were upregulated by an ABA treatment (Figure 5A), but only the promoters of *MdSultr3;1a, MdSultr3;1b, MdSultr3;1c, MdSultr3;1d, MdSultr3;3a*, and *MdSultr3;3b* contained ABREs (Figure 2D), which is the major cis-element for ABA crosstalk with various stressors (Chen et al., 2020b). Except for *MdSultr3;3b* and *MdSultr4;2*, which were upregulated, the other *MdSultrs* were downregulated under the IAA treatment (Figure 5B), however, IAA-responsive elements were only found in the promoters of *MdSultr3;1a, MdSultr3;1c, MdSultr3;5*, and *MdSultr4;2* (Figure 2D), which is consistent with previous studies in potatoes (Vatansever et al., 2016). Furthermore, the expression of *MdSultr3;1d, MdSultr3;3b*, and *MdSultr3;5b* was significantly upregulated by the MeJA treatment (Figure 5C), while MeJA-responsive elements were found in the promoters of these genes (Figure 2D). Plant hormones play a critical role in regulating plant growth, plant development, and primary metabolism, including ${\text{SO}}_{4}^{2-}$ uptake and assimilation (Koprivova and Kopriva, 2016). Considering the presence of hormone-responsive elements in the promoter region of *MdSultrs* (Figure 2D), together with the response of *MdSultrs* to hormones (Figure 5), *MdSultrs* may act as key mediators of hormone-induced changes in S metabolism, which requires further in-depth study.

The expression of sulfate transporters is induced by low S treatment. The expression of *MdSultr3;1a, MdSultr3;1b, MdSultr3;1c*, and *MdSultr3;1d* was rapidly upregulated in *Malus* roots within a short period of a low-S treatment (Figure 4A), in which *MdSultr3;1a* and *MdSultr3;1d* were strongly expressed in the roots (Figure 3B). This is consistent with a study reporting that *AtSultr1;1* in Arabidopsis and *LeST1-1* in tomato, which was mainly expressed in the roots and upregulated by S starvation, were primarily responsible for the root uptake of ${\text{SO}}_{4}^{2-}$ from the soil (Takahashi et al., 2000; Yoshimoto et al., 2002; Howarth et al., 2003). In contrast, *ZmSultr3;1* was also specifically expressed in maize roots but was not affected by S deficiency (Huang et al., 2018); similarly, the expression of Group 3 Sultrs in wheat was not affected by S deficiency (Buchner et al., 2010). These results suggest that Sultr genes exhibit different expression patterns from others in the same Group 3, indicating possible functional divergence among the species.

In Arabidopsis, *AtSultr1;1* and *AtSultr1;2* were specifically expressed in root hairs, and the epidermal and cortical cells regulated ${\text{SO}}_{4}^{2-}$ uptake by the roots (Takahashi et al., 2000; Yoshimoto et al., 2002). *MdSultr3;1a* showed root-specific expression (Figure 3B) and was significantly upregulated by low S (Figure 4A). *MhSultr3;1a* was highly homologous to *MdSultr3;1a*, and its protein localized at the cell plasma and nuclear membranes (Figure 6), indicating that *MhSultr3;1a* may be involved in ${\text{SO}}_{4}^{2-}$ uptake at the root/soil interface under S-limiting conditions. *MhSultr3;1a* was able to complement the yeast mutant CP154-7A, which lacked ${\text{SO}}_{4}^{2-}$ uptake capability when grown on a YNB medium containing 0.1 mM of MgSO_4_ as the sole source of S (Figure 7A). However, the Group 3 genes in Arabidopsis and *OsSultr3;3* in rice failed to complement the defect of the mutant (Takahashi et al., 2000; Kataoka et al., 2004a; Zhao et al., 2016), presumably because they may not have been correctly recognized as plasma membrane-localizing protein in yeast cells (Takahashi et al., 2000). However, in Arabidopsis, five of the genes in Group 3 were localized in the chloroplast and were involved in ${\text{SO}}_{4}^{2-}$ uptake and transport in the chloroplast (Cao et al., 2013; Chen et al., 2019). *OsSultr3;3*, localized in the endoplasmic reticulum (Zhao et al., 2016), was involved in ${\text{SO}}_{4}^{2-}$ homeostasis, metabolism, and partitioning processes (Zhao et al., 2016). Similarly, the overexpression of *MhSultr3;1a* in apple calli accumulated higher ${\text{SO}}_{4}^{2-}$ content (Figure 8A). These results suggest that Sultr exhibits species specificity. Moreover, several studies confirmed that the overexpression of Sultr genes could improve plant growth under low-S conditions by upregulating the genes involved in the S assimilation pathway and promoting the biosynthesis of essential amino acids and S-containing compounds (Ding et al., 2016; Narayan et al., 2021). In our study, under low-S conditions, the overexpression of *MhSultr3;1a* in apple calli resulted in better growth (Figure 7C) and higher fresh weight (Figure 7D). This may be because the accumulation of ${\text{SO}}_{4}^{2-}$ (Figure 8A) and Cys (Figure 8B) contents enhanced the biosynthesis of essential amino acids and S-containing compounds required for growth. Additional research is necessary to understand the function of Sultrs in *Malus* and its regulatory mechanisms involved in growth and hormone responsiveness.

## Conclusion

**Figure 9:**
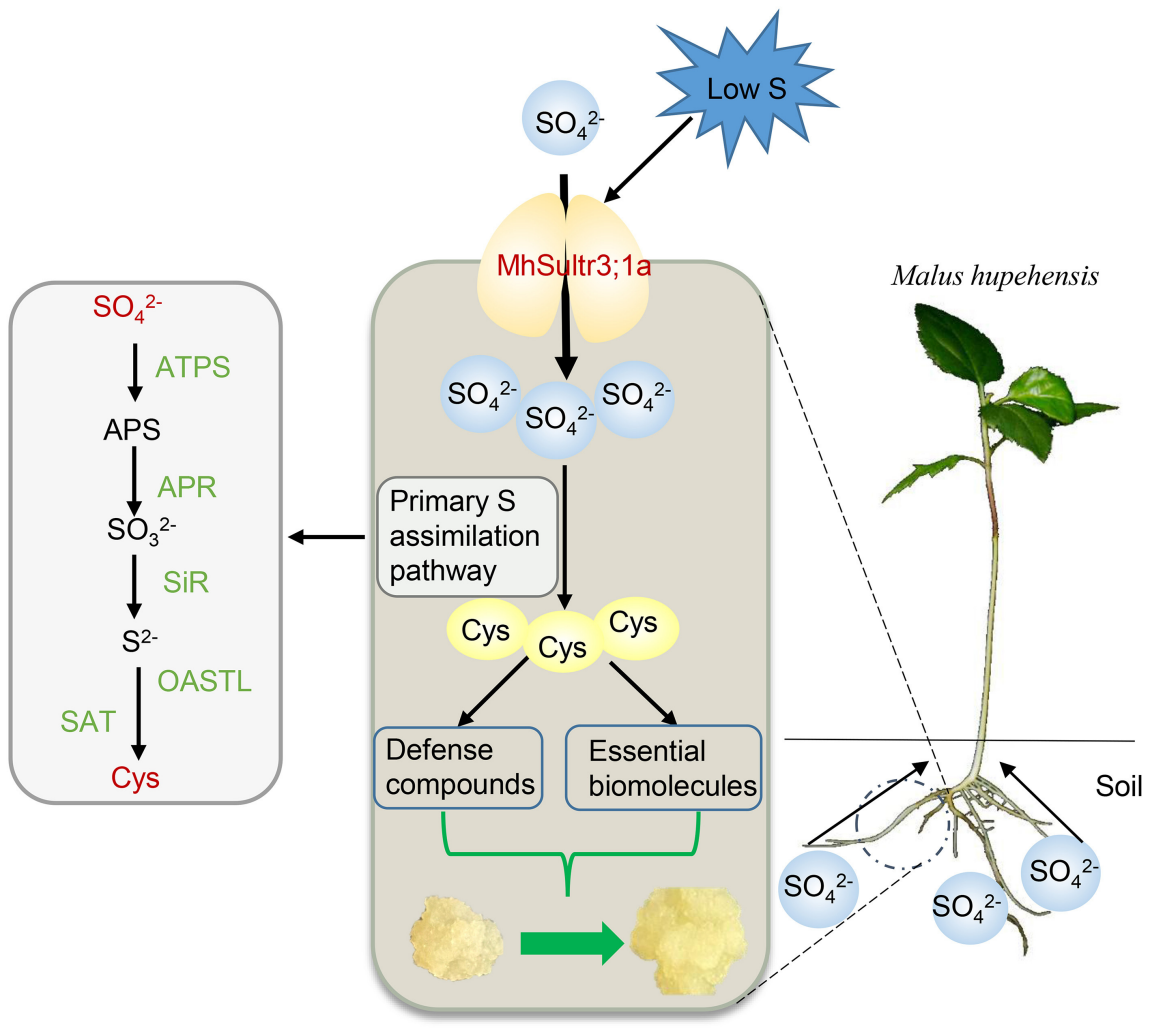
Proposed role of *MhSultr3;1a* in improving growth under low-S conditions. *MhSultr3;1a* was responsible for the uptake of ${\text{SO}}_{4}^{2-}$ from the soil by *Malus* roots and induced by a low-S treatment. Furthermore, the upregulation of *MhSultr3;1a* accumulated more ${\text{SO}}_{4}^{2-}$ into Cys *via* the primary S assimilation pathway. Cys is a precursor for the synthesis of essential biomolecules and defense compounds, which is essential for the improvement of growth under S-limiting conditions. ATPS, ATP sulfurylase; APR, APS reductase; SiR, sulfite reductase; OASTL, OAS (thiol)-lyase; SAT, serine acetyltransferase.

In this study, nine *MdSultrs* genes were identified in the *M. domestica* genome and divided into two subfamilies. An analysis of the phylogenetic tree, gene structures, and protein motifs revealed that *MdSultr* proteins are conserved and have the typical features of Sultrs. The expression of apple *MdSultrs* was tissue-specific and induced by low S and hormones such as ABA and MeJA. *MhSultr3;1a* encoded a functional Sultr and increased ${\text{SO}}_{4}^{2-}$ uptake and Cys content to meet the demand for S-containing compounds under S-limiting conditions, improving growth (Figure 9).

## Data Availability Statement

The datasets presented in this study can be found in online repositories. The names of the repository/repositories and accession number(s) can be found below: https://www.ncbi.nlm.nih.gov/genbank/, MZ634458.

## Author Contributions

MX and HY designed the experiments. JFS helped in the analysis of the genome-wide identification of *MdSultrs*. JYS, YS, JY, and JL helped perform the experiments and analyze the data. MX and WZ wrote the manuscript. HY revised the manuscript and served as the project leader. All authors read and approved its final version.

## Funding

This work was supported by the National Key R&D Program of China (No. 2019YFD1000103) and the National Natural Science Foundation of China (No. 31772251).

## Conflict of Interest

The authors declare that the research was conducted in the absence of any commercial or financial relationships that could be construed as a potential conflict of interest.

## Publisher's Note

All claims expressed in this article are solely those of the authors and do not necessarily represent those of their affiliated organizations, or those of the publisher, the editors and the reviewers. Any product that may be evaluated in this article, or claim that may be made by its manufacturer, is not guaranteed or endorsed by the publisher.

## References

[B1] Aarabi F. Naake T. Fernie A. R. Hoefgen R. (2020). Coordinating sulfur pools under sulfate deprivation. Trends Plant Sci. 25, 1227–1239. 10.1016/j.tplants.2020.07.007 32800669

[B2] Akbudak M. A. Filiz E. Kontbay K. (2018). Genome-wide identification and cadmium induced expression profiling of sulfate transporter (SULTR) genes in sorghum (*Sorghum bicolor* L.). Biometals 31, 91–105. 10.1007/s10534-017-0071-5 29236185

[B3] Al Murad M. Razi K. Benjamin L. K. Lee J. H. Kim T. H. Muneer S. (2020). Ethylene regulates sulfur acquisition by regulating the expression of sulfate transporter genes in oilseed rape. Physiol. Plantar. 171, 533–545. 10.1111/ppl.13157 32588442

[B4] An J. P. Qu F. J. Yao J. F. Wang X. N. You C. X. Wang X. F.. (2017). The bZIP transcription factor MdHY5 regulates anthocyanin accumulation and nitrate assimilation in apple. Hortic. Res. 4, 17023. 10.1038/hortres.2017.23 28611922 PMC5461414

[B5] Batool S. Uslu V. V. Rajab H. Ahmad N. Waadt R. Geiger D.. (2018). Sulfate is incorporated into cysteine to trigger ABA production and stomatal closure. Plant Cell 30, 2973–2987. 10.1105/tpc.18.00612 30538155 PMC6354274

[B6] Buchner P. Parmar S. Kriegel A. Carpentier M. Hawkesford M. J. (2010). The sulfate transporter family in wheat: tissue-specific gene expression in relation to nutrition. Mol. Plant 3, 374–389. 10.1093/mp/ssp119 20118181

[B7] Buchner P. Stuiver C. E. Westerman S. Wirtz M. Hell R. Hawkesford M. J.. (2004a). Regulation of sulfate uptake and expression of sulfate transporter genes in Brassica oleracea as affected by atmospheric H_2_S and pedospheric sulfate nutrition. Plant Physio. 136, 3396–3408. 10.1104/pp.104.046441 15377780 PMC523398

[B8] Buchner P. Takahashi H. Hawkesford M. J. (2004b). Plant sulphate transporters: co-ordination of uptake, intracellular and long-distance transport. J. Exp. Bot. 55, 1765–1773. 10.1093/jxb/erh206 15258169

[B9] Cao M. J. Wang Z. Wirtz M. Hell R. Oliver D. J. Xiang C. B. (2013). SULTR3;1 is a chloroplast-localized sulfate transporter in *Arabidopsis thaliana*. Plant J. 73, 607–616. 10.1111/tpj.12059 23095126

[B10] Chen C. J. Chen H. Zhang Y. Thomas H. R. Frank M. H. He Y. H.. (2020a). TBtools: an integrative toolkit developed for interactive analyses of big biological data. Mol. Plant 13, 1194–1202. 10.1016/j.molp.2020.06.009 32585190

[B11] Chen K. Li G. J. Bressan R. A. Song C. P. Zhu J. K. Zhao Y. (2020b). Abscisic acid dynamics, signaling, and functions in plants. J. Integr. Plant Biol. 62, 25–54. 10.1111/jipb.12899 31850654

[B12] Chen T. Tian M. Han Y. (2020c). Hydrogen sulfide: a multi-tasking signal molecule in the regulation of oxidative stress responses. J. Exp. Bot. 71, 2862–2869. 10.1093/jxb/eraa093 32076713

[B13] Chen X. S. Li T. T. Zhou S. L. Zhao Y. (2018). Transient expression of exogenous protein in tobacco leaves. Bio Protocol. 10.21769/BioProtoc.1010127

[B14] Chen Z. Zhao P. X. Miao Z. Q. Qi G. F. Wang Z. Yuan Y.. (2019). SULTR3s function in chloroplast sulfate uptake and affect ABA biosynthesis and the stress response. Plant Physiol. 180, 593–604. 10.1104/pp.18.01439 30837346 PMC6501079

[B15] Cherest H. Davidian J. C. Thomas D. Benes V. Ansorge W. Surdin-Kerjan Y. (1997). Molecular characterization of two high affinity sulfate transporters in Saccharomyces cerevisiae. Genetics 145, 627–635. 9055073 10.1093/genetics/145.3.627 PMC1207848

[B16] Ding Y. Q. Zhou X. Q. Zuo L. Wang H. Yu D. Y. (2016). Identification and functional characterization of the sulfate transporter gene GmSULTR1;2b in soybean. BMC Genom. 17:373. 10.1186/s12864-016-2705-3 27206527 PMC4874011

[B17] Dürr J. Bücking H. Mult S. Wildhagen H. Palme K. Rennenberg H.. (2010). Seasonal and cell type specific expression of sulfate transporters in the phloem of Populus reveals tree specific characteristics for ${\text{SO}}_{4}^{2-}$ storage and mobilization. Plant Mol. Biol. 72, 499–517. 10.1007/s11103-009-9587-6 20087755

[B18] Edgar R. C. (2004). MUSCLE: multiple sequence alignment with high accuracy and high throughput. Nucleic Acids Res. 32, 1792–1797. 10.1093/nar/gkh340 15034147 PMC390337

[B19] Gigolashvili T. Kopriva S. (2014). Transporters in plant sulfur metabolism. Front. Plant Sci. 5:442. 10.3389/fpls.2014.00442 25250037 PMC4158793

[B20] Howarth J. R. Fourcroy P. Davidian J. C. Smith F. W. Hawkesford M. J. (2003). Cloning of two contrasting high-affinity sulfate transporters from tomato induced by low sulfate and infection by the vascular pathogen *Verticillium dahliae*. Planta 218, 58–64. 10.1007/s00425-003-1085-5 12937983

[B21] Huang Q. Wang M. Xia Z. (2018). The SULTR gene family in maize (Zea mays L.): gene cloning and expression analyses under sulfate starvation and abiotic stress. J. Plant Physiol. 220, 24–33. 10.1016/j.jplph.2017.10.010 29145069

[B22] Kataoka T. Hayashi N. Yamaya T. Takahashi H. (2004a). Root-to-shoot transport of sulfate in Arabidopsis. Evidence for the role of SULTR3;5 as a component of low-affinity sulfate transport system in the root vasculature. Plant Physiol. 136, 4198–4204. 10.1104/pp.104.045625 15531709 PMC535849

[B23] Kataoka T. Watanabe-Takahashi A. Hayashi N. Ohnishi M. Mimura T. Buchner P.. (2004b). Vacuolar sulfate transporters are essential determinants controlling internal distribution of sulfate in Arabidopsis. Plant Cell 16, 2693–2704. 10.1105/tpc.104.023960 15367713 PMC520965

[B24] Kopriva S. Malagoli M. Takahashi H. (2019). Sulfur nutrition: impacts on plant development, metabolism, and stress responses. J. Exp. Bot. 70, 4069–4073. 10.1093/jxb/erz319 31423538

[B25] Koprivova A. Kopriva S. (2016). Hormonal control of sulfate uptake and assimilation. Plant Mol. Biol. 91, 617–627. 10.1007/s11103-016-0438-y 26810064

[B26] Kulczycki G. (2021). The effect of elemental sulfur fertilization on plant yields and soil properties. Adv. Agron. 167, 105–181. 10.1016/bs.agron.2020.12.003

[B27] Kumar S. Asif M. H. Chakrabarty D. Tripathi R. D. Trivedi P. K. (2011). Differential expression and alternative splicing of rice sulphate transporter family members regulate sulphur status during plant growth, development and stress conditions. Funct. Integr. Genom. 11, 259–273. 10.1007/s10142-010-0207-y 21221698

[B28] Kumar S. Khare R. Trivedi P. K. (2019). Arsenic-responsive high-affinity rice sulphate transporter, OsSultr1;1, provides abiotic stress tolerance under limiting sulphur condition. J. Hazard. Mater. 373, 753–762. 10.1016/j.jhazmat.2019.04.011 30965240

[B29] Kumar S. Stecher G. Tamura K. (2016). MEGA7: molecular evolutionary genetics analysis Version 7.0 for bigger datasets. Mol. Biol. Evol. 33, 1870–1874. 10.1093/molbev/msw054 27004904 PMC8210823

[B30] Lancilli C. Giacomini B. Lucchini G. Davidian J. C. Cocucci M. Sacchi G. A.. (2014). Cadmium exposure and sulfate limitation reveal differences in the transcriptional control of three sulfate transporter (Sultr1;2) genes in Brassica juncea. BMC Plant Biol. 14:132. 10.1186/1471-2229-14-132 24884748 PMC4049391

[B31] Liu D. D. Dong Q. L. Sun C. Wang Q. L. You C. X. Yao Y. X.. (2012). Functional characterization of an apple apomixis-related MhFIE gene in reproduction development. Plant Sci. 185–186, 105–111. 10.1016/j.plantsci.2011.09.004 22325871

[B32] Maruyama-Nakashita A. Nakamura Y. Yamaya T. Takahashi H. (2004). A novel regulatory pathway of sulfate uptake in Arabidopsis roots: implication of CRE1/WOL/AHK4-mediated cytokinin-dependent regulation. Plant J. 38, 779–789. 10.1111/j.1365-313X.2004.02079.x 15144379

[B33] Narayan O. P. Verma N. Jogawat A. Dua M. Johri A. K. (2021). Sulfur transfer from the endophytic fungus Serendipita indica improves maize growth and requires the sulfate transporter SiSulT. Plant Cell 33, 1268–1285. 10.1093/plcell/koab006 33793849

[B34] Rajab H. Khan M. Malagoli M. Hell R. Wirtz M. (2019). Sulfate-induced stomata closure requires the canonical ABA signal transduction machinery. Plants 8:21. 10.3390/plants8010021 30654485 PMC6359059

[B35] Romero L. C. Aroca M. Á. Laureano-Marín A. M. Moreno I. García I. Gotor C. (2014). Cysteine and cysteine-related signaling pathways in Arabidopsis thaliana. Mol. Plant 7, 264–276. 10.1093/mp/sst168 24285094

[B36] Shi W. Liu W. Ma C. Zhang Y. Ding S. Yu W.. (2020). Dissecting MicroRNA-mRNA regulatory networks underlying sulfur assimilation and cadmium accumulation in Poplar leaves. Plant Cell Physiol. 61, 1614–1630. 10.1093/pcp/pcaa084 32678905

[B37] Shibagaki N. Grossman A. R. (2006). The role of the STAS domain in the function and biogenesis of a sulfate transporter as probed by random mutagenesis. J. Biol. Chem. 281, 22964–22973. 10.1074/jbc.M603462200 16754669

[B38] Shibagaki N. Grossman A. R. (2010). Binding of Cysteine Synthase to the STAS Domain of Sulfate Transporter and Its Regulatory Consequences. J. Biol. Chem. 285, 25094–25102. 10.1074/jbc.M110.126888 20529854 PMC2915745

[B39] Sun T. T. Li M. J. Shao Y. Yu L. Y. Ma F. W. (2017). Comprehensive genomic identification and expression analysis of the Phosphate Transporter (PHT) gene family in Apple. Front. Plant Sci. 8:426. 10.3389/fpls.2017.00426 28424713 PMC5371654

[B40] Takahashi H. (2010). Regulation of sulfate transport and assimilation in plants. Int. Rev. Cell Mol. Biol. 281, 129–159. 10.1016/s1937-6448(10)81004-4 20460185

[B41] Takahashi H. (2019). Sulfate transport systems in plants: functional diversity and molecular mechanisms underlying regulatory coordination. J. Exp. Bot. 70, 4075–4087. 10.1093/jxb/erz132 30907420

[B42] Takahashi H. Watanabe-Takahashi A. Smith F. W. Blake-Kalff M. Hawkesford M. J. Saito K. (2000). The roles of three functional sulphate transporters involved in uptake and translocation of sulphate in *Arabidopsis thaliana*. Plant J. 23, 171–182. 10.1046/j.1365-313x.2000.00768.x 10929111

[B43] Vatansever R. Koc I. Ozyigit I. I. Sen U. Uras M. E. Anjum N. A.. (2016). Genome-wide identification and expression analysis of sulfate transporter (SULTR) genes in potato (*Solanum tuberosum* L.). Planta 244, 1167–1183. 10.1007/s00425-016-2575-6 27473680

[B44] Yamaji N. Takemoto Y. Miyaji T. Mitani-Ueno N. Yoshida K. T. Ma J. F. (2017). Reducing phosphorus accumulation in rice grains with an impaired transporter in the node (vol 541, pg 92, 2017). Nature 541, 136–136. 10.1038/nature21404 28002408

[B45] Yang H. Q. Duan K. X. Zhang W. W. (2008). Biology and physiology of Malus hupehensis for the apogamic plant resource. Acta Hortic. 769, 441–447. 10.17660/ActaHortic.2008.769.63

[B46] Yang H. Q. Fan W. W. (2012). Advances in research of apple root system architecture and it's regulation. Acta Hortic. Sinica 39, 1673–1678. 10.16420/j.issn.0513-353x.2012.09.010

[B47] Yoshimoto N. Inoue E. Saito K. Yamaya T. Takahashi H. (2003). Phloem-localizing sulfate transporter, Sultr1;3, mediates re-distribution of sulfur from source to sink organs in Arabidopsis. Plant Physiol. 131, 1511–1517. 10.1104/pp.014712 12692311 PMC166910

[B48] Yoshimoto N. Takahashi H. Smith F. W. Yamaya T. Saito K. (2002). Two distinct high-affinity sulfate transporters with different inducibilities mediate uptake of sulfate in Arabidopsis roots. Plant J. 29, 465–473. 10.1046/j.0960-7412.2001.01231.x 11846879

[B49] Zhang H. J. Hao X. Y. Zhang J. J. Wang L. Wang Y. C. Li N. N.. (2021). Genome-wide identification of SULTR genes in tea plant and analysis of their expression in response to sulfur and selenium. Protoplasma. 1–14. 10.1007/s00709-021-01643-z 33884505

[B50] Zhang W. W. Yue S. Q. Song J. F. Xun M. Han M. Y. Yang H. Q. (2020). MhNRAMP1 from Malus hupehensis exacerbates cell death by accelerating Cd uptake in tobacco and apple calli. Front. Plant Sci. 11:957. 10.3389/fpls.2020.00957 32733509 PMC7358555

[B51] Zhao H. J. Frank T. Tan Y. Y. Zhou C. G. Jabnoune M. Arpat A. B.. (2016). Disruption of OsSULTR3;3 reduces phytate and phosphorus concentrations and alters the metabolite profile in rice grains. New Phytol. 211, 926–939. 10.1111/nph.13969 27110682

