# Supplementary material for: Genome-Wide Identification of Sultr Genes in Malus domestica and Low Sulfur-Induced MhSultr3;1a to Increase Cysteine-Improving Growth
Source: Front Plant Sci. 2021 Oct 11;12:748242. doi: 10.3389/fpls.2021.748242 (PMC8544799; doi:10.3389/fpls.2021.748242)
Supplement: Supplementary file 1 [file Data_Sheet_1.docx]

Supplementary Figures


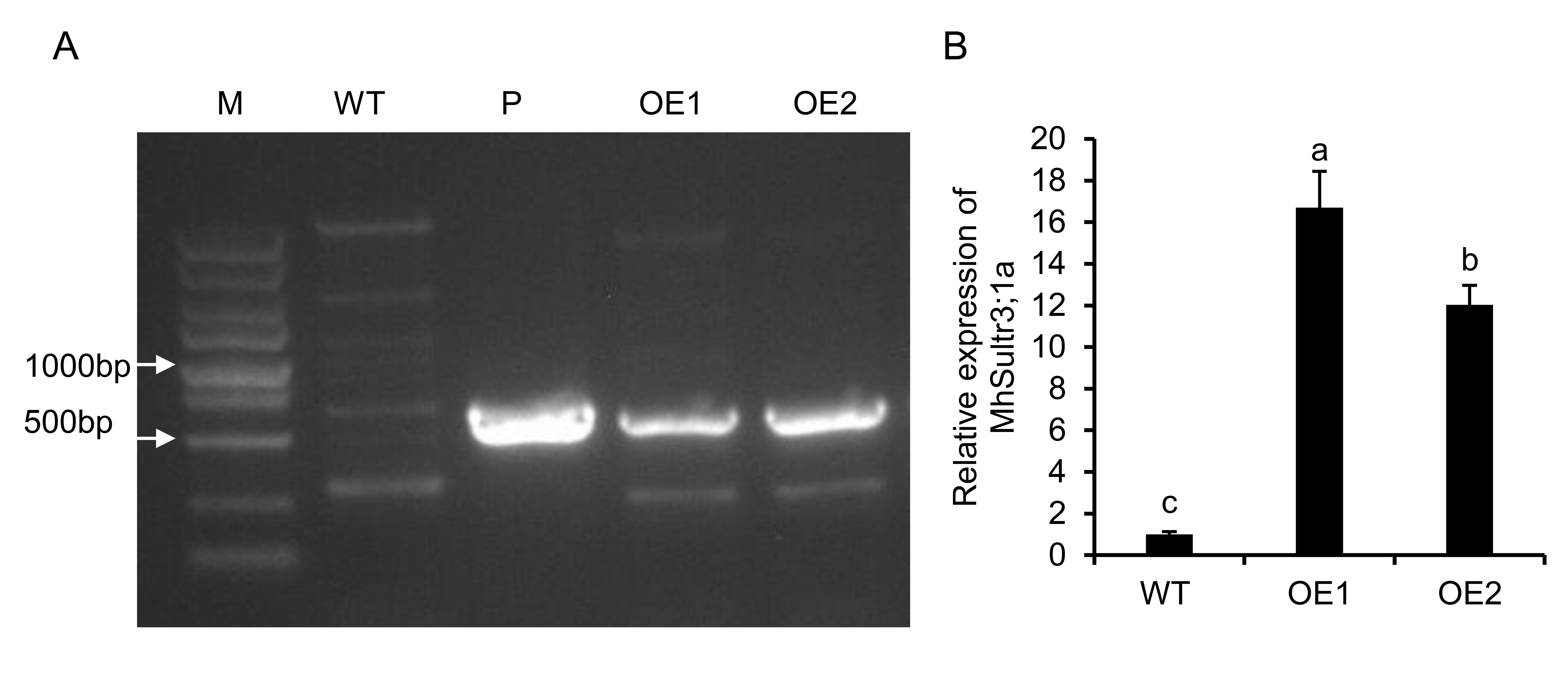


**Supplementary Figure 1** (A) gDNA-PCR analysis of MhSultr3;1a in wild type (WT) and overexpression transgenic apple calli lines (OE1 and OE2). M means DL5000 DNA marker, WT was as negative control, P means pGWB405-MhSultr3;1a expression vector, which was as positive control. (B) qRT-PCR analysis of expression levels of MhSultr3;1a in WT, OE1 and OE2. Data are presented as the mean ± SD of three independent biological replicates. Different letters above the columns indicate significant differences (*p<0.05*).





**Supplementary Figure 2 The sequence analysis and subcellular localization of *MhSultr3;1a*.** Multiple alignment (A) and phylogenetic analysis (B) of MhSultr3;1a in *Malus hupehensis* and several Sultr3;1s proteins from other plants, including *Pyrus bretschneideri*，*Prunus avium*，*Prunus persica*，*Populus trichocarpa* and *Arabidopsis thaliana*. Multiple sequence alignments were performed using DNAMAN. Neighbor-joining (NJ) phylogenetic tree was contrusted using MEGA 7.0 with 1000 bootstrap replications, passion model, and pairwise deletion.
