# Supplementary material for: Genome-Wide Identification of Sultr Genes in Malus domestica and Low Sulfur-Induced MhSultr3;1a to Increase Cysteine-Improving Growth
Source: Front Plant Sci. 2021 Oct 11;12:748242. doi: 10.3389/fpls.2021.748242 (PMC8544799; doi:10.3389/fpls.2021.748242)
Supplement: Supplementary file 2 [file Data_Sheet_2.docx]

**Supplementary Tables**

**Supplementary Table 1 GenBank accession numbers of the proteins used for phylogenetic analysis** **and** **multiple sequence alignments**

| Protein name | GenBank Accession number |
| --- | --- |
| AT4G08620 (AtSultr1;1) | NP_192602.1 |
| AT1G78000.1 (AtSultr1;2) | NP_001321366.1 |
| AT1G22150.1(AtSultr1;3) | NP_001319061.1 |
| AT5G10180.1(AtSultr2;1) | NP_196580.1 |
| AT1G77990.1(AtSultr2;2) | NP_565165.2 |
| AT3G51895.1(AtSultr3;1) | NP_190758.2 |
| AT4G02700.1(AtSultr3;2) | NP_192179.1 |
| AT1G23090.1(AtSultr3;3) | NP_173722.1 |
| AT3G15990.1(AtSultr3;4) | NP_188220.1 |
| AT5G19600.1(AtSultr3;5) | NP_568377.1 |
| AT5G13550.1(AtSultr4;1) | NP_196859.1 |
| AT3G12520.1(AtSultr4;2)  MdSultr3;1a  MdSultr3;1b  MdSultr3;1c  MdSultr3;1d  MdSultr3;3a  MdSultr3;3b  MdSultr3;4  MdSultr3;5  MdSultr4;2 | NP_187858.1  XP_008390997.2  XP_008390930.1  XP_008368821.2  XP_008366542.1  RXH70282.1  XP_008389211.3  TQE04443.1  RXH74574.1  XP_008371139.1 |
| MhSultr3;1a  PaSultr3;1  PbSuLTR3;1  PpSultr3;1  PtSultr3;1 | MZ634458  XP_021828165.1  XP_009371954.1  XP_007225118.1  XP_002314667.2 |

**Supplementary Table 2 The primers used for cloning, subcellular localization, and vector construction of *MhSultr3;1a***

| Primer name | Primer sequence(5’-3’) |
| --- | --- |
| MhSultr3;1a-F | ATGGGCAACGCAGATTATGA |
| MhSultr3;1a-R | AACATTGCCATCTTGTCTGTTTG |
| MhSultr3;1a-EF  MhSultr3;1a-ER | GGGGACAAGTTTGTACAAAAAAGCAGGCTTCATGGGCAACGCAGATTATGA  GGGGACCACTTTGTACAAGAAAGCTGGGTTAACATTGCCATCTTGTCTGTTTG |
| MhSultr3;1a-YF | ctgctgcagtctagagaattcATGGGCAACGCAGATTATGAG |
| MhSultr3;1a-YR | aagaagtccaaagctggatccAACATTGCCATCTTGTCTGTTTGT |

**Supplementary Table 3 The primers used for confirming transgenic apple calli**

| Primer name | Primer sequence(5’-3’) | Used for |
| --- | --- | --- |
| NeoR-F | CTATTCGGCTATGACTGGGC | gDNA-PCR |
| NeoR-R  MhSultr3;1a  MhSultr3;1a | AATATCACGGGTAGCCAACG  GGAATCACCATTGCCAGTCT  GCTAACAACCTTCCCCAACA | qRT-PCR |

**Supplementary Table 4 The primers for qRT-PCR of *MdSultr* genes**

| Gene ID | Gene name | Forward primes | Reverse primes |
| --- | --- | --- | --- |
| MDP0000085223 | MdSultr3;1a | GGAATCACCATTGCCAGTCT | GCTAACAACCTTCCCCAACA |
| MDP0000216466 | MdSultr3;1b | TTTGTGCCTCCATTGGTGTA | AAGTCCACGACAAACCCAAG |
| MDP0000231619 | MdSultr3;1c | TTCCTGATGACCCTTTGAGG | CCAAGAATTGGTGGCAAGTT |
| MDP0000311618 | MdSultr3;1d | GCGGAGTTTTGGGTTGTTTA | AGGTCTCCAAACGACAATGG |
| MDP0000145668 | MdSultr3;3a | ACTGGCATTATTGCCCTCAC | TTCGACATTGCTGTCTTTGC |
| MDP0000190006 | MdSultr3;3b | AAGGAGTGGTCATGGCAAAC | TGAAAAACCAGCATGTTCCA |
| MDP0000317974 | MdSultr3;4 | TTTCCCAGACAACCCACTTC | CCAACAATTGGAGGCAAACT |
| MDP0000167489 | MdSultr3;5 | CTCCACTTTTCGTGGTGGTT | AGTAATAGCCGCAGCCTTCA |
| MDP0000141450 | MdSultr4;2 | AGGCCTGGTGGACTATGATG | ATATGCGGGTTTGCTGATTC |
|  | Md-actin | TAAGGCTGGATTTGCTGGAG | GCATCTTTCTGACCCATTCC |

**Supplementary Table 5 List of putative motifs of *MdSultr* proteins**

| Motif | Conservative sequence | Width | Pfam domain |
| --- | --- | --- | --- |
| 1 | EGIAVGRSFAAFKNYHIDGNKEMIAIGMMNIVGSCTSCYLTTGPFSRSAV | 50 | Sulfate_transp (PF00916) |
| 2 | MVTLLFLTPLFHYTPLVVLSAIIITAMLGLIDYEAAIHLWKVDKYDFLVC | 50 | Sulfate_transp (PF00916) |
| 3 | LKETFFPDDPLRQFKNQPPSRKLILGLQYFFPILEWAPRYT | 41 | Not found |
| 4 | MKSVFSQRHEWRWESAVLGCCFLFFLLLTRYFSKKKPKLFWISAMAPLVS | 50 | Sulfate_transp (PF00916) |
| 5 | PSASNIPGVLILQIDAPIYFANTNYLRERILRWIYEEEDRIKS | 43 | STAS (PF01740) |
| 6 | PQGISYAKLANLPPIIGLYSSFVPPLIYA | 29 | Sulfate_transp (PF00916) |
| 7 | LAFTATFFAGIFQASLGLLRLGFIVDFLSHATIVGFMAGAATVVCLQQLK | 50 | Sulfate_transp (PF00916) |
| 8 | FLTHAEKHGIQVIGHLKKGJNPPSFNDLVFGSPYLATAIKTGIITGIIAL | 50 | Sulfate_transp (PF00916) |
| 9 | RYVILDMSAVATIDTSGITMLEEVRKSIDKRGJKLVJANPRGEVMKKLQK | 50 | STAS (PF01740) |
| 10 | GVVFISVZIGLAIAVGISLFRVLLYVARPRTFVLGNIPGSSIYRNIDQY | 49 | Not found |
| 11 | MLGSSKDLAVGTVAVASLLIASMLGEEVS | 29 | Sulfate_transp (PF00916) |
| 12 | NYNAGAKTAMSNIVM | 15 | Not found |
| 13 | WIYLTVAEAVAACSFMLHTTK | 21 | Not found |
| 14 | KSDJISGITIASLAI | 15 | Not found |
| 15 | EESPHRVEIPPPQPFIKVLKS | 21 | Not found |
| 16 | LGJKHFTHKTDVVSV | 15 | Not found |
| 17 | EBPTLYLQ | 8 | Not found |
| 18 | DCCSPTRINIZEQQP | 15 | Not found |
